# Autonomous Polymer Frameworks for Sustainable Tissue‐Interfaced Plastic Bioelectronics

**DOI:** 10.1002/advs.202515320

**Published:** 2025-11-29

**Authors:** Elvis K. Boahen, Zhengyang Kong, So Young Kim, Hayoung Oh, Hanseo Yoo, Jeong Sub Lim, Hyun Joon Shin, Ji Hong Kim, Do Hwan Kim

**Affiliations:** ^1^ Department of Chemical Engineering Hanyang University Seoul 04763 Republic of Korea; ^2^ Institute of Nano Science and Technology Hanyang University Seoul 04763 Republic of Korea; ^3^ Clean‐Energy Research Institute Hanyang University Seoul 04763 Republic of Korea

**Keywords:** autonomous polymer frameworks, plastic bioelectronics, sustainable electronics, tissue‐interfaced applications

## Abstract

Recent advancements in polymer science have enabled the development of plastic bioelectronics, providing soft, stretchable, and tissue‐conformable technologies for continuous health monitoring, diagnostics, and therapeutic interventions. Unlike conventional silicon‐based electronics that often exhibit mechanical mismatches with biological tissues, plastic bioelectronic systems leverage intrinsically soft and mechanically compliant organic and polymer materials to achieve enhanced conformability. This reduces interfacial stress and enables high‐fidelity signal acquisition from dynamic tissue interfaces. However, the low mechanical modulus that enables their unique advantages also makes these systems susceptible to mechanical damage, weak adhesion, and functional degradation under physiological conditions. To overcome these limitations, emerging research focuses on integrating autonomous polymer frameworks (auto‐POFs)–engineered materials that endow the polymer matrix with self‐adhesion, self‐protection, self‐healing, self‐degradation, and self‐sensing capabilities. These features enable real‐time responsiveness to stimuli and extend device lifespan without external intervention. This review provides a comprehensive overview of recent progress in auto‐POF‐based systems, including their material design strategies, functional mechanisms, and roles in enhancing the reliability and adaptability of sustainable, wearable, and implantable tissue‐interfaced plastic bioelectronics. By highlighting key material innovations and device architectures, the path is outlined toward next‐generation biomedical platforms capable of autonomous and sustainable operation in dynamic biological environments.

## Graphical abstract

This review focuses on plastic bioelectronic systems integrated with autonomous polymer frameworks (auto‐POFs) and summarizes recent progress in this field. It provides an in‐depth analysis of the design strategies and operational mechanisms of auto‐POFs, highlighting their pivotal role in enhancing the sustainability and interfacial adaptability of bioelectronic systems, and outlines the future direction for the development of next‐generation wearable and implantable systems.

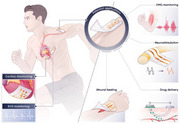

## 1 Introduction

Plastic bioelectronics is a research field that has been increasingly adopted in healthcare for the development of smart, intelligent, and wearable biomedical systems, owing to their lightweight nature, flexibility, and inherently stretchable properties.^[^ ^1^, ^2^, ^3^, ^4^ ^]^ This platform refers to functional electronic devices that exhibit the mechanical characteristics of plastics.^[^ ^5^, ^6^, ^7^, ^8^ ^]^ By leveraging the intrinsic properties of polymers and organic materials, these devices enable seamless integration of electronics with biological tissues and organs, offering distinct advantages over traditional silicon (Si)‐based electronics.^[^ ^9^, ^10^, ^11^, ^12^, ^13^, ^14^, ^15^ ^]^ Conventional Si‐based medical electronics are primarily composed of rigid materials with extremely high Young's modulus values, typically ranging from 100 to 300 GPa.^[^ ^16^, ^17^ ^]^ In contrast, biological tissues and organs exhibit much lower moduli, generally between 0.1 kPa and 2 MPa.^[^ ^16^, ^18^, ^19^, ^20^, ^21^ ^]^ This substantial mechanical mismatch often results in limited conformability, increased interfacial stress, and poor adhesion at the tissue‐device interface, thereby compromising sensing accuracy, physiological monitoring fidelity, and the effectiveness of therapeutic interventions.^[^ ^21^, ^22^ ^]^ Moreover, such mismatches can induce adverse biological responses, including irritation, inflammation, or even tissue damage.^[^ ^23^, ^24^, ^25^, ^26^ ^]^ Although reducing the thickness of rigid Si‐based components may improve flexibility to some extent, it fails to fully resolve the modulus mismatch problem.^[^ ^16^, ^27^ ^]^ In contrast, plastic bioelectronic systems utilize intrinsically soft and compliant materials with low Young's moduli, typically between 1 kPa to 10 GPa,^[^ ^28^, ^29^ ^]^ offering superior conformability and mechanical compatibility with soft tissues. These characteristics enable the fabrication of wearable and implantable devices that can seamlessly integrate with biological systems, allowing for real‐time acquisition of physiological signals and targeted therapeutic interventions for persona**lized heal**thcare applications.^[^ ^30^, ^31^, ^32^ ^]^

Recent advancements in plastic bioelectronic technologies have significantly broadened the scope of this field, enabling the development of next‐generation sustainable electronic systems that are not only stretchable and biodegradable but also self‐adaptive.^[^ ^33^, ^34^, ^35^, ^36^, ^37^ ^]^ These features are particularly beneficial for applications such as continuous health monitoring, transient medical implants, and neural interfaces designed for targeted biomedical functions. Due to their low mechanical moduli, these systems can form conformal interfaces with biological tissues, thereby minimizing irritation or tissue damage while enhancing the accuracy and precision of bioelectrical signal acquisition, which are critical parameters for both wearable and implantable medical devices.^[^ ^38^, ^39^, ^40^ ^]^

Despite these advantages, the intrinsic low mechanical modulus that imparts high deformability, flexibility, and stretchability also renders plastic bioelectronic devices susceptible to several limitations. These may include: 1) unexpected mechanical damage caused by continuous stress or tear;^[^ ^41^ ^]^ 2) weak interfacial adhesion with tissues, which may lead to delamination under moist conditions and result in low signal‐to‐noise ratios (SNR) during signal acquisition;^[^ ^31^ ^]^ and 3) degradation of electrical performance due to environmental factors such as moisture absorption, oxidation, and exposure to biofluids.^[^ ^42^, ^43^ ^]^ Collectively, these challenges lead to diminished device performance, reduced signal fidelity, ineffective therapeutic outcomes, and ultimately, shortened device lifespan.^[^ ^44^, ^45^ ^]^ Therefore, a major materials design challenge lies in engineering plastic bioelectronic systems that maintain low Young's modulus for optimal mechanical compliance with soft tissues while simultaneously ensuring long‐term durability and functionality under physiological conditions.

**Figure 1:**
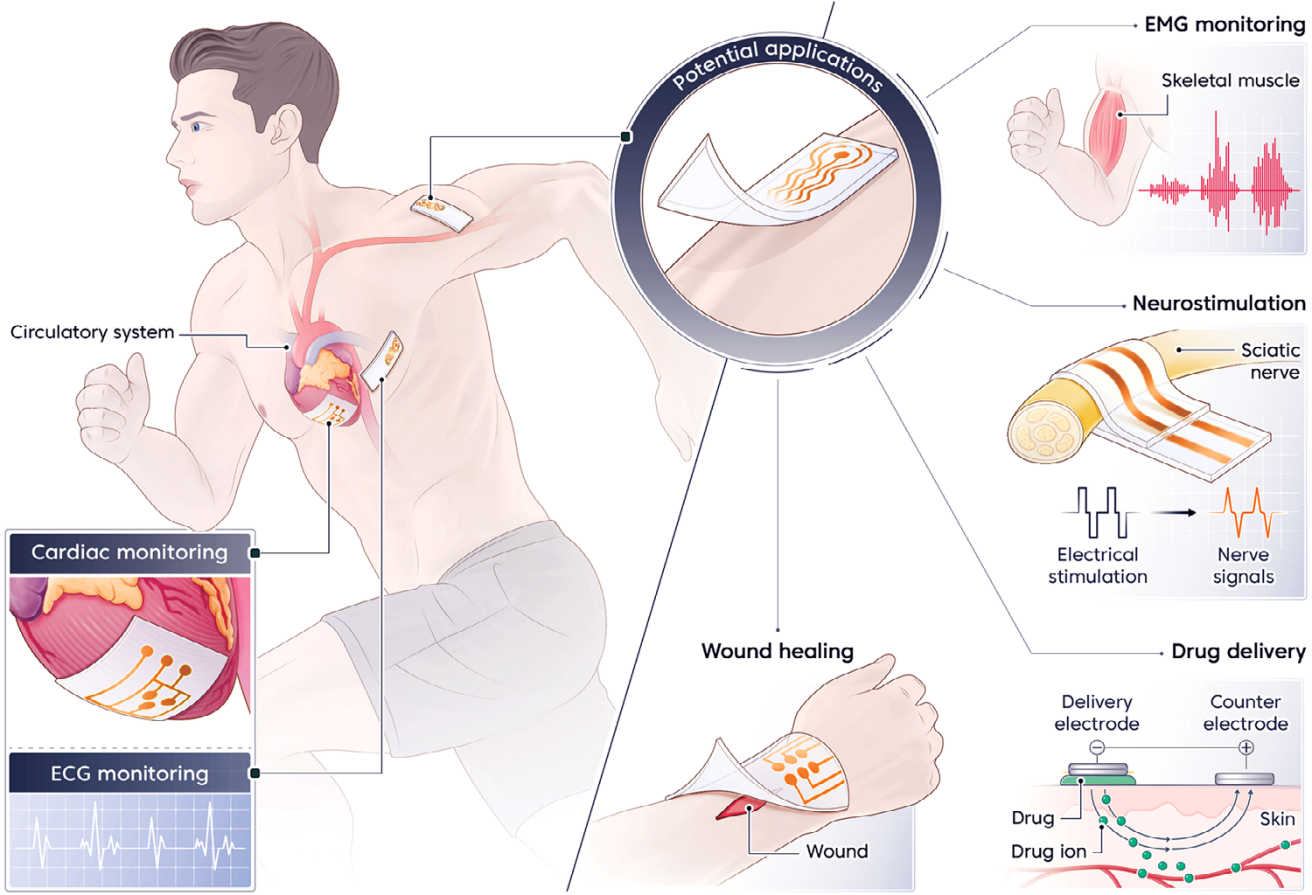
Tissue‐interfaced plastic bioelectronic systems. Structural illustration showing the integration of tissue‐interfaced plastic bioelectronic systems across diverse biomedical applications. Representative functionalities include cardiac monitoring, electrophysiological sensing (ECG and EMG), neurostimulation, controlled drug delivery, and wound healing, highlighting the adaptability of plastic bioelectronics in real‐time health monitoring and therapeutic interventions.

To address these limitations, recent research has focused on integrating autonomous polymer frameworks (auto‐POFs) into plastic bioelectronic materials and devices. These frameworks incorporate intrinsic functionalities, such as self‐adhesion, self‐protection, self‐healing, self‐degradation, and self‐sensing to facilitate robust and seamless integration at the tissue‐device interface. Auto‐POFs represent an emerging class of advanced polymeric materials engineered to impart adaptive and self‐regulating capabilities to polymers and organic systems.^[^ ^46^, ^47^ ^]^ These built‐in modalities are specifically designed to mitigate performance degradation under harsh environmental or physiological conditions, thereby enhancing operational stability, prolonging device lifespan, and supporting the development of sustainable electronic technologies. Plastic bioelectronic systems integrated with auto‐POFs can autonomously sense, respond to, or adapt to dynamic environmental cues in real time.^[^ ^47^, ^48^ ^]^ This marks a significant advancement over conventional stimuli‐responsive polymers, which typically rely on external triggers, such as ultraviolet light or temperature changes, to activate functions like healing or degradation.^[^ ^49^ ^]^ Such dependence on external stimuli may pose risks to surrounding tissues or cells, potentially causing irritation, inflammation, or disruption of physiological processes, thereby limiting their clinical applicability.^[^ ^50^, ^51^ ^]^ Therefore, the incorporation of auto‐POF modalities into plastic bioelectronic systems is essential for enabling independent, stimuli‐free operation–ensuring safer, more effective, and autonomous device function in real‐world biomedical environments. As illustrated in **Figure** **1**, plastic bioelectronics holds considerable promise for a wide range of clinical applications, including electrophysiological and biophysical signal monitoring, diagnosis of cardiovascular‐related diseases, chronic wound healing, controlled drug delivery, and neurostimulation. These technological advances position plastic bioelectronics as a transformative platform for sustainable health monitoring, diagnostics, and therapeutics across various biomedical domains.

In this review, we provide a comprehensive overview of recent progress in the development of auto‐POFs‐integrated sustainable plastic bioelectronic systems for tissue‐interfaced biomedical applications, with a particular emphasis on wearable (on‐skin) and implantable platforms. We specifically focus on three representative categories–bio‐adhesives, life‐control functions, and sensory applications–because they directly embody the intrinsic autonomous modalities of auto‐POFs and illustrate how these functionalities address critical challenges (such as delamination, mechanical fracture, and signal drift) in tissue‐device integration, long‐term durability, and real‐time responsiveness. By organizing the discussion around these categories, we aim to clarify how individual autonomous functions can be tailored and combined to meet the requirements of practical biomedical devices. We begin by defining and contextualizing the foundational concept of auto‐POFs, followed by a critical analysis of their integration into plastic bioelectronic systems. We then explore key molecular‐ and device‐level strategies for tailoring auto‐POF modalities to achieve optimal biomedical performance. Finally, we conclude with perspectives on future directions and some of the remaining challenges in advancing auto‐POF technologies within the broader framework of sustainable plastic bioelectronics. Through this review, we aim not only to elucidate the emerging concept of auto‐POFs but also to underscore their transformative potential for next‐generation bioelectronic technologies.

## 2 Autonomous Polymer Frameworks

Biological systems employ intrinsic capabilities to autonomously sense, respond to, and adapt to prevailing environmental conditions.^[^ ^52^, ^53^ ^]^ For example, human bone tissue possesses the ability to sense and regulate mechanical stress by adjusting its density, either by thickening or thinning in response to varying levels of stress.^[^ ^54^, ^55^ ^]^ Additionally, bones can exhibit a remarkable ability to self‐heal upon injury, restoring their functionality within the biological setting.^[^ ^55^, ^56^, ^57^, ^58^ ^]^ Inspired by these biological mechanisms and driven by advancements in polymer science and engineering, researchers have developed auto‐POFs that replicate these self‐sustained and autonomous functionalities observed in nature.^[^ ^47^, ^59^, ^60^ ^]^ Auto‐POFs refer to bioinspired polymer systems engineered to sense, respond to, or adapt in real‐time to environmental conditions, without the need for external stimuli or intervention.^[^ ^47^, ^61^ ^]^ These systems rely on intrinsic properties like viscoelasticity,^[^ ^62^, ^63^, ^64^ ^]^ chemical reactivity,^[^ ^65^ ^]^ as well as environmental responsiveness^[^ ^66^ ^]^ to perform specific tasks independently. For instance, auto‐POFs can self‐regulate, self‐heal, or modulate their inherent properties in response to changes in mechanical deformation, temperature, or other physiological conditions.^[^ ^67^, ^68^ ^]^ These adaptive characteristics most often occur in real‐time and are crucial for ensuring long‐term, reliable performance of plastic bioelectronic systems, particularly in tissue‐interfaced applications, where continuous functionality is essential without requiring periodic external intervention. The integration of auto‐POFs into tissue‐interfaced plastic bioelectronics represents a paradigm shift in biomedical systems, enabling materials that autonomously adjust their properties to align with the dynamic and ever‐changing environment of biological tissues and organs.

**Figure 2:**
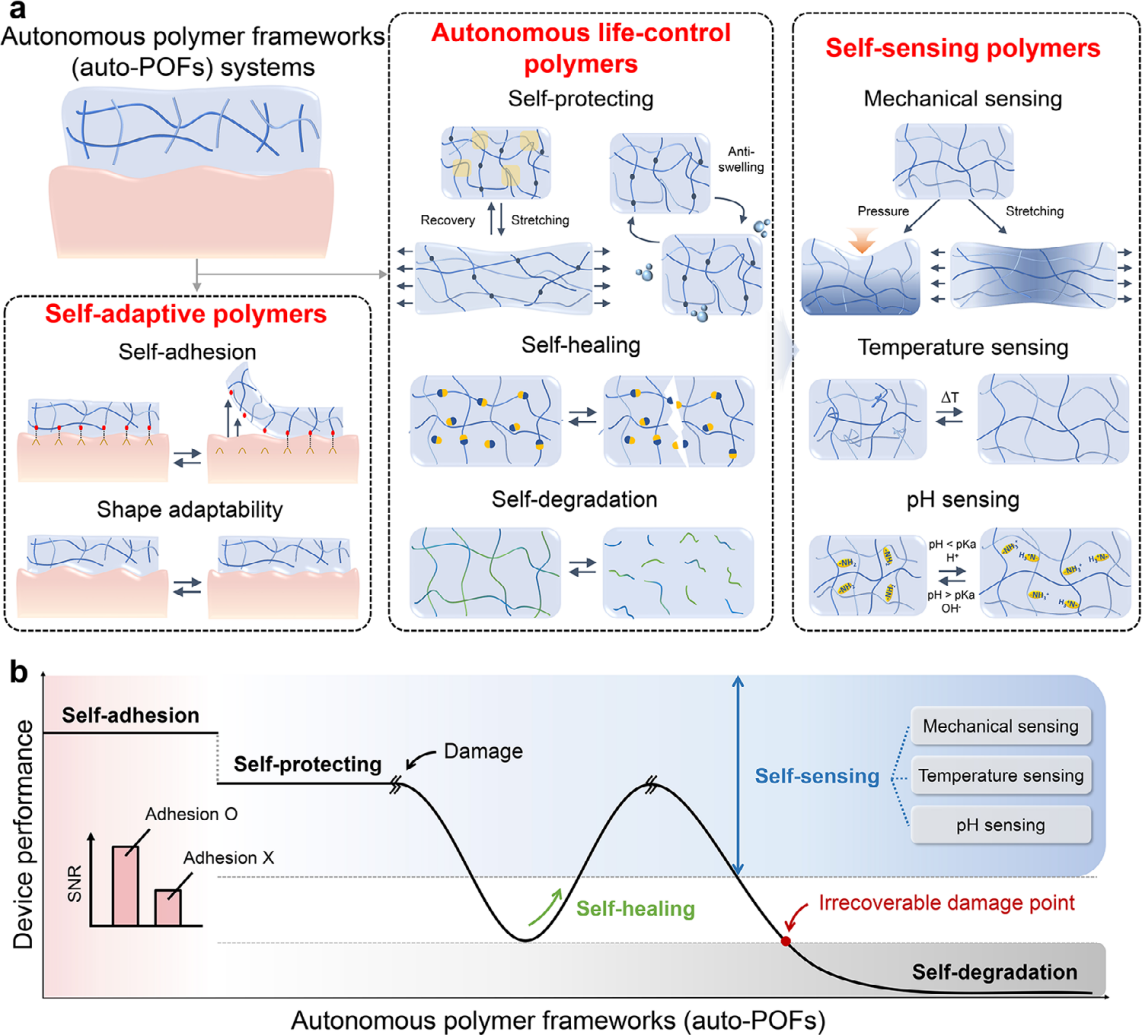
The concept of autonomous polymer frameworks. a) Schematic overview of key autonomous polymer framework (auto‐POF) modalities that enable plastic bioelectronic systems to autonomously maintain and restore functionality. These modalities can be grouped into three functional classes: self‐adaptive polymers (e.g., self‐adhesion), autonomous life‐control polymers (e.g., self‐protection, self‐healing, and self‐degradation), and self‐sensing polymers. The self‐adaptive class includes not only self‐adhesion, but also stimuli‐responsive modulation (e.g., drug release triggered by pH, temperature, or enzymatic activity) and mechanical adaptation (e.g., real‐time adjustment of stiffness or compliance to match tissue dynamics). Both stimuli‐responsive modulation and mechanical adaptation primarily influence the material's mechanical integrity, particularly its conformal tissue integration under dynamic physiological conditions. b) A conceptual chart illustrating how integration of auto‐POF modalities sustains device performance across different stages of the operational lifecycle. The *x*‐axis (“auto‐POFs”) denotes sequential autonomous functionalities (self‐adhesion, self‐protection, self‐healing, and self‐degradation) while the *y*‐axis (“device performance”) represents relative functional reliability and stability. Self‐adhesion enhances initial tissue contact and signal quality (SNR improvement); self‐protection mitigates environmental and mechanical stress during operation; self‐healing restores structural and electrical integrity after damage; and self‐degradation ensures controlled disintegration at the end of service life. Together, these modalities enable resilient and sustainable operation of tissue‐interfaced plastic bioelectronic systems.

**Figure** **2**a illustrates some of the standard auto‐POF modalities that can enable plastic bioelectronic systems to maintain and restore their functional capabilities. These modalities can be broadly categorized into three functional classes: self‐adaptive polymers (e.g., self‐adhesion), autonomous life‐control polymers, and self‐sensing polymers. We note that the term “life‐control polymers” is employed here to describe systems capable of autonomously governing their full operational lifecycle–including self‐protection, self‐healing, and programmed self‐degradation–rather than merely modulating properties. This terminology emphasizes the concept of complete lifecycle regulation, which is critical for sustainable bioelectronic operation. This classification highlights the modular nature of auto‐POFs and their complementary roles in supporting sustainable device operation across a range of biomedical applications.

Self‐adaptivity enables polymers to autonomously adjust interfacial, mechanical, or transport properties in response to internal or environmental cues, thereby ensuring consistent performance in dynamic biological conditions. A representative modality is self‐adhesion, which ensures intimate and persistent contact between bioelectronic devices and biological tissues, thereby enhancing SNR by eliminating the need for external adhesives, heat, or chemical treatments.^[^ ^69^ ^]^ Such adhesion is achieved through robust, reversible intermolecular interactions such as hydrogen bonding, van der Waals forces, and electrostatic interactions, which promote reliable bonding even under physiologically dynamic conditions.^[^ ^70^, ^71^, ^72^ ^]^ Beyond adhesion, self‐adaptivity also encompasses broader functional mechanisms, including: i) the suppression of sensing noise and drift through real‐time mechanical compliance that stabilizes tissue‐device interfaces; ii) cue responsive drug delivery, where therapeutic payloads are released in response to physicochemical or biochemical cues‐such as pH fluctuations, enzyme mediated degradation, or temperature variations; and iii) dynamic modulation or reconfiguration of adhesion strength under varying wet or mechanical loading conditions.

Self‐protection safeguards against potentially damaging environmental conditions, including mechanical strain, stress, and swelling in high‐humidity environments.^[^ ^73^ ^]^ This protective effect can be achieved by incorporating crosslinking density and/or hydrophobic groups, which form a physical barrier that maintains the functionality of the polymer even under mechanical deformation (e.g., stretching, bending, or piercing), as well as regulating moisture and fluid ingress.^[^ ^39^ ^]^

In the event of unexpected mechanical damage, self‐healing mechanisms enable the polymer to repair microcracks or other forms of physical damage in real time, restoring both structural and functional integrity without the need for external stimuli.^[^ ^74^ ^]^ For example, microcrack formation within a self‐healing conductive polymer can transiently increase resistance, which is then autonomously restored upon dynamic bond reformation, thereby recovering both structural integrity and electrical conductivity. This process is facilitated by the introduction of microcapsules^[^ ^75^ ^]^ or incorporation of inherent factors such as polymer chain mobility and reversible dynamic bonds,^[^ ^59^, ^60^, ^76^ ^]^ which can break and reform via dynamic covalent or non‐covalent interactions.

When the polymer reaches the end of its operational life or experiences irreversible damage, self‐degradation becomes essential. This process involves breaking down the polymer into smaller non‐toxic components that are environmentally friendly or can be absorbed by the bloodstream in the case of implantable devices.^[^ ^77^ ^]^ The degradation process may occur through mechanisms such as oxidation,^[^ ^78^, ^79^ ^]^ hydrolysis,^[^ ^80^ ^]^ or enzymatic activity,^[^ ^81^ ^]^ depending on the chemical structure of the polymer and the environmental conditions in which it operates.

Additionally, self‐sensing functionality enables autonomous detection and response in real time to various environmental stimuli without the need for external sensor modules. These stimuli include mechanical deformations (such as pressure and strain), temperature fluctuations, pH variations, or the presence of specific biological analytes.^[^ ^82^ ^]^ When such external stimuli are applied, they can induce reversible changes in the material's internal microstructure, molecular conformation, or chemical composition, thereby modulating its electrical properties, such as conductivity, resistance, and capacitance. The resulting electrical signals can be collected and output in real time, enabling the immediate transduction and feedback of environmental inputs. On this basis, these self‐sensing capabilities allow for the construction of intelligent interfaces capable of local signal acquisition and transmission without additional signal processing units, significantly simplifying system architecture and offering a promising strategy for autonomous signal detection and response in bioelectronic devices.

Finally, Figure 2b illustrates how these autonomous functionalities support distinct stages of the device performance lifecycle. It is important to note that Figure 2b does not depict a single device, but rather a conceptual framework showing how sequential auto‐POF modalities–self‐adhesion, self‐protection, self‐healing, and self‐degradation–collectively sustain device performance throughout its operational lifecycle. Self‐adhesion enhances initial signal quality by ensuring intimate and stable tissue contact, while self‐protection mitigates mechanical and environmental stresses during operation. In the event of damage, self‐healing restores both structural and functional integrity, and upon irreversible failure, self‐degradation facilitates controlled material disintegration. Collectively, these staged responses enable adaptive and resilient operation of tissue‐interfaced plastic bioelectronic systems without the need for external intervention. These enable the development of plastic bioelectronic systems capable of performing complex tasks autonomously. As a result, these systems are particularly well‐suited for the dynamic environments encountered in tissue‐interfaced bioelectronics, offering more reliable and versatile solutions for sustainable applications ranging from wearable health‐monitoring devices to neural interface systems.^[^ ^28^, ^74^, ^77^ ^]^

## 3 Self‐Adaptive Interfacial Bioadhesive Systems for Tissue‐Interfaced Bioelectronics

Building upon the expanded definition of self‐adaptivity, this section highlights interfacial self‐adaptive systems‐particularly bioadhesive polymers that facilitate stable and dynamic coupling between bioelectronic devices and biological tissues. In tissue‐interfaced bioelectronics, achieving reliable adhesion under wet, irregular, and continuously moving conditions remains a major challenge. Maintaining such conformal and robust contact is critical for ensuring long‐term signal fidelity, minimizing motion artifacts, and preserving therapeutic efficacy. Bioadhesives have therefore been widely employed in applications ranging from wound closure and implant fixation to wearable electronics, where stable tissue‐device interfaces are essential for accurate physiological sensing and diagnostics.^[^ ^83^, ^84^ ^]^ However, conventional adhesives often fall short under dynamic physiological conditions (particularly in vivo) where tissue surfaces are continuously hydrated and mechanically active. It is therefore important to establish conformal, durable, and stable contact between bioelectronic devices and target tissues. Bioadhesives enable strong adhesion to diverse, dynamic, and wet biological tissues and are used across applications such as adjunctive closure and sealing of wounds and surgical incisions, skin attachment of wearable devices, and localized fixation of implantable devices. For bioelectronic interfaces, reliable tissue adhesion is essential for stable signal acquisition and precise diagnostics.^[^ ^83^, ^84^ ^]^

**Figure 3:**
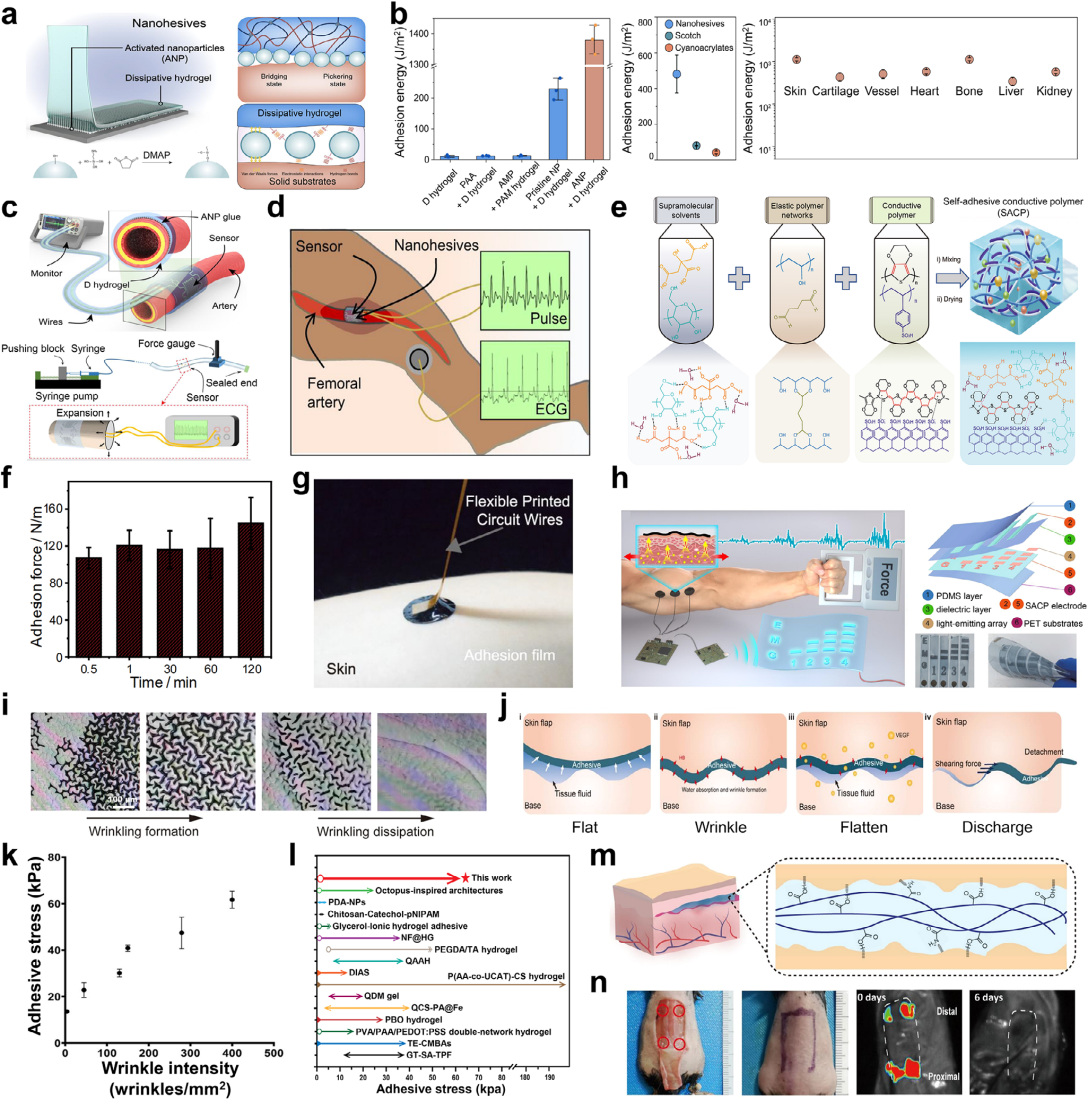
Self‐adaptive bioadhesive polymer‐based systems for tissue‐interface bioelectronics. a) Schematic illustration of the nanohesive design and silanization process of silica nanoparticles. b) Evaluation of nanohesive adhesion strength on various substrates and biological tissues. c) Arterial pulsation monitoring setup and the underlying blood signal detection mechanism. d) Schematics depicting blood flow monitoring on the femoral artery of a canine. a–d) Reproduced with permission.^[^ ^85^ ^]^ Copyright 2023, Springer Nature. e) SACP fabrication process, chemical structures, and key interactions, including covalent crosslinking, electrostatic interactions, and supramolecular solvent effects. f) Relationship between bonding strength with time. g) Illustration of adhesion between epidermis and flexible printed wire interconnects. h) ACEL display system is controlled by EMG sensors, where patterned lighting arrays are integrated with a transparent conductive film. e–h) Reproduced with permission.^[^ ^86^ ^]^ Copyright 2022, Springer Nature. i) Wrinkle formation upon hydration and dissipation after immersion in water. j) Mechanism of skin adhesion and detachment of the film triggered by tissue fluid exudation. k) Correlation between the degree of surface wrinkling and adhesive performance. l) Summary of reported adhesive strengths of previously studied switchable bioadhesives. m) Adhesion of the film to skin under stretching, twisting, and bending. n) Skin flap model surgery and in vivo fluorescence imaging showing film adhesive. i–n) Reproduced with permission.^[^ ^89^ ^]^ Copyright 2023, American Association for the Advancement of Science.

In view of these requirements, recent studies have introduced self‐adhesive, autonomously switchable bioadhesive polymer platforms that enable robust wet‐surface adhesion and on‐demand, reversible attachment and detachment while preserving tissue‐like mechanics. However, achieving conformal, durable contact in vivo remains challenging due to irregular, hydrated, and constantly moving surfaces.^[^ ^85^ ^]^ To address this challenge, a novel class of adhesives, known as “nanohesives,” have recently been designed to enable fast and robust adhesion of hydrogels to diverse surfaces, including engineering materials and biological tissues, without requiring any surface pretreatments.^[^ ^85^ ^]^ These nanohesives were fabricated from surface‐activated nanoparticles (ANP) modified with carboxyl groups, which were integrated into a dissipative hydrogel network (**Figure** **3**a). The carboxyl modification of ANP increased adhesion sites, while the hydrogel network was prepared using a long‐chain covalently and physically crosslinked components for robust adhesion. The adhesion mechanism was based on a synergistic interaction between the ANP and the hydrogel network. In wet environments, the hydrogel rapidly absorbed interfacial moisture, facilitating the condensation of the ANP at the adhesive interfaces (Figure 3a). This resulted in the formation of strong interfacial bonds through short‐range interactions, including van der Waals interactions, hydrogen bonding, and electrostatic interactions, enabling fast adhesion within seconds. The nanohesives exhibited high interfacial adhesion energies ranging from 500 to 1400 Jm^−2^, allowing for strong adhesion to a wide range of materials such as metals, ceramics, plastics, rubbers, and various biological tissues (Figure 3b). In practical application, the nanohesives were employed to attach strain sensors onto blood vessels, enabling real‐time, in vivo monitoring of blood flow signals in a canine femoral artery with high reliability and accuracy (Figure 3c,d). Although the nanohesive exhibited exceptional adhesion performance to various materials and biological tissues in both wet and dynamic environments, they served solely as a biocompatible glue and lack inherent sensing capability. A vital criterion to enable continuous adaptation to varying environmental conditions, such as strain, pressure, or temperature changes, without the need for additional sensor integration. Moreover, the integration of additional sensors introduces device complexity. Therefore, developing a self‐adaptive bioadhesive with integrated inherent sensing functionality is highly desired.

As shown in Figure 3e, a self‐adhesive conductive polymer (SACP) composite based on a solution‐processable blend of Poly(3,4‐ethylenedioxythiophene):poly(styrene sulfonate) (PEDOT:PSS), a biocompatible supramolecular solvent (SMS) composed of β‐cyclodextrin and citric acid, and poly(vinyl alcohol) (PVA) covalently crosslinked with glutaraldehyde (GA) has been developed.^[^ ^86^ ^]^ This composite material demonstrated several advantages, including a low modulus (56.1–401.9 kPa), high interfacial adhesion strength (lap‐shear strength >1.2 MPa), high conductivity (1–37 S cm^−1^), and high stretchability (700%). The incorporation of the SMS introduced a high density of carboxyl and hydroxyl groups, which reduced the aggregation of PEDOT chains. SMS supplies a high density of –COOH/–OH groups that engage in multidentate hydrogen bonding and form electrostatic interactions with PSS and positively charged PEDOT. These interactions suppress PEDOT interchain aggregation, thereby improving chain mobility and interfacial compliance. Concurrently, the abundant hydroxyl and charged species generate multiple, cooperative weak bonds at the substrate interface, increasing the real contact area and interfacial energy.^[^ ^87^ ^]^ Collectively, this SMS‐enabled interfacial chemistry accounts for the rapid and strong adhesion observed across diverse substrates, including biological skin (Figure 3f,g). Moreover, the PVA covalently crosslinked with GA formed an elastic network that imparts reversible stretchability to the SACP composite material. Additionally, owing to its high electrical conductivity and low electrical impedance, the SACP‐based device was capable of effectively capturing bioelectrical signals generated by muscle contractions, ensuring efficient signal transmission for electromyography (EMG) monitoring (Figure 3h). Thus, this study not only successfully achieved high conductivity and low modulus, simultaneously within a PEDOT:PSS‐based composite, but also enabled robust tissue adhesion.

Despite the distinctive advantages of the aforementioned bioadhesive systems in biomedical applications, their strong adhesion properties often result in tissue injury or inflammation upon detachment at the end of their operational life.^[^ ^88^ ^]^ To ensure both stable adhesion and injury‐free detachment, researchers have developed an autonomous switchable hydrogel film with programmable tissue adhesion and detachment, based on reversible surface wrinkling patterns (Figure 3i).^[^ ^89^ ^]^ This design concept was inspired by natural structures such as the feet of gecko lizards and beetles, which utilize dynamic surface topology (including wrinkling and buckling), to modulate adhesion and release.^[^ ^90^, ^91^ ^]^ By leveraging bilayer buckling dynamics, a self‐similar hierarchical wrinkling morphology was engineered in the hydrogel film composed of a copolymer of acrylic acid (AA) and acrylamide (AM).^[^ ^89^ ^]^ This composition enables hydration‐responsive, reversible wrinkling patterns for the regulation of adhesion strength. The AA introduced carboxylic acid groups that facilitated hydrogen bonding and electrostatic interactions, and the poly(AM) endowed the film with amide groups for both enhanced adhesion and hydration‐induced swelling that drives the wrinkling formation. In an initial dry state, the film adhered strongly to the tissue interface through dense wrinkle formation induced by compressive stress in the bilayer system, resulting in conformal contact and strong interfacial adhesion via hydrogen bonds. Upon exposure to tissue fluid, AA absorbed interfacial water, causing the hydrogel to swell and increasing the density of hydrogen bonds, thereby enhancing adhesion. However, as the film absorbed more fluid, the wrinkles flattened, decreasing the compressive stress and consequently lowering the adhesion strength. This transition enabled spontaneous, injury‐free detachment from the tissue (Figure 3j). This switchable adhesion mechanism demonstrated superior adhesive performance compared to previously reported switchable bioadhesives (Figure 3k,l). To demonstrate its practical functionality, the hydrogel film was used for tissue sealing in a mouse dorsal skin flap model (Figure 3m,n). The adhesive securely sealed the wound margins and subsequently detached autonomously as it absorbed tissue fluids. This facilitated sufficient effective tissue sealing while avoiding secondary tissue injury commonly associated with the removal of conventional strong adhesives. To address these removal‐related risks, it is necessary to look beyond chemistry alone and consider how the interface behaves across scales. Growing evidence indicates that adhesion arises from a coupled hierarchy in which molecular interactions provide interfacial affinity, while the real (effective) contact area and the management of interfacial water, which regulate hydration layers, swelling‐induced modulus changes, and wrinkling‐mediated topography, ultimately set the usable adhesion window.

The self‐adaptive bioadhesive systems discussed in this section are capable of autonomously forming strong and stable bonds with various surfaces without requiring surface pretreatments or external interventions. Additionally, the integration of autonomous programmable functionalities with bioadhesives makes these adhesives highly favorable for both on‐skin and implantation biomedical applications.

## 4 Autonomous Life‐Control of Plastic Bioelectronic Polymers

The operational lifespan of conventional plastic bioelectronic materials is primarily limited by mechanical wear and tear, environmental factors, and unexpected damage.^[^ ^92^ ^]^ These factors ultimately lead to performance degradation, failure, and eventual disposal. In contrast, plastic bioelectronic materials integrated with auto‐POFs emulate the intrinsic functionalities of biological systems by autonomously achieving self‐protection, self‐healing, and self‐degradation.^[^ ^93^ ^]^ These autonomous behaviors can be enabled through various material design strategies. For instance, enhancing mechanical robustness and incorporating hydrophobic groups can improve resistance to tearing and swelling, respectively.^[^ ^94^ ^]^ Furthermore, embedding self‐healing functionalities can allow materials to recover from unexpected mechanical damage,^[^ ^95^ ^]^ while integrating self‐degradable capabilities ensures safe disposal after usage.^[^ ^96^ ^]^ Collectively, these capabilities enhance material durability, extend device lifespan, and contribute to sustainability in biomedical applications. Multiple approaches have been explored to implement these functionalities in plastic bioelectronic systems.^[^ ^1^ ^]^ In the following section, we examine some of the recent advances in the development of self‐protecting, self‐healing, and self‐degrading polymer materials specifically tailored for tissue‐interfaced bioelectronic applications.

### 4.1 Self‐Protecting Polymers for Tissue‐Interfaced Bioelectronics

**Figure 4:**
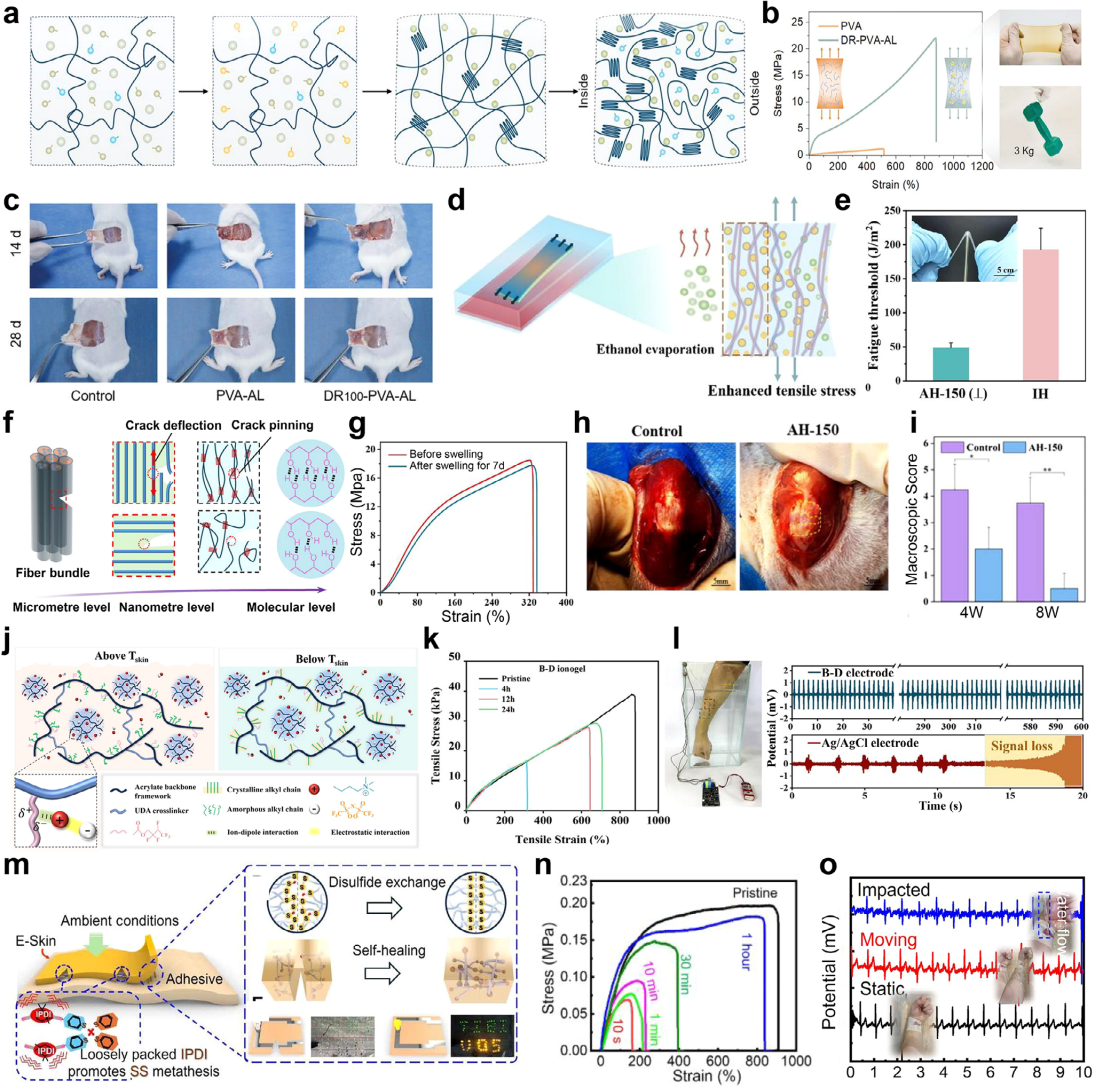
Self‐protecting and autonomous self‐healing polymers for tissue‐interfaced bioelectronics. a) Schematic illustration of the self‐protective DR‐PVA‐AL hydrogel prepared via a multi‐solvent high‐temperature annealing process. Folded dark blue chains denote crystalline domains; olive green circles denote lignin; sky blue circles with tails denote water molecules; olive green circles with tails denote DMSO molecules; and orange circles with tails denote ethanol molecules. b) Tensile behavior of PVA and DR100‐PVA‐AL hydrogels, with corresponding optical images of the latter. c) Photograph showing DR‐PVA‐AL hydrogel implantation into rat tissue as a biomedical substitute material after 14 and 24 days. a–c) Reproduced with permission.^[^ ^101^ ^]^ Copyright 2025, Wiley‐VCH. d) Schematic depicting macromolecular chain aggregation and alignment induced by poor solvent evaporation. e) Anti‐swelling performance of AHs after 7 days in water, with a summary of corresponding fatigue thresholds for IH and AH‐150. f) Crack tolerance mechanism of AHs across three different scales: micrometer, nanometer, and molecular levels. g) Representative tensile stress–strain curves of AH‐150 before and after 7‐day water exposure. h,i) Demonstration of rat tendon repair using AHs following injury. d–i) Reproduced with permission.^[^ ^102^ ^]^ Copyright 2025, Wiley‐VCH. j) Diagram illustrating the microstructure of the ionogel and the phase transition behavior of its long alkyl side chains. k) Representative stress–strain curves of the original and self‐healed ionogels after different healing durations. l) Stability of underwater EMG signals recorded using ionogel and Ag/AgCl electrodes. j–l) Reproduced with permission.^[^ ^118^ ^]^ Copyright 2025, Wiley‐VCH. m) Schematics and optical images showing disulfide bond‐mediated self‐healing of the E‐skin, enabled by IPDI incorporation into the TPU matrix. Functional recovery is demonstrated by LED relighting after complete severing. n) Mechanical profile of the E‐skin during progressive healing following full incision. o) Underwater ECG signals under static, dynamic, and water‐impact conditions, with corresponding images. m–o) Reproduced with permission.^[^ ^122^ ^]^ Copyright 2025, American Association for the Advancement of Science.

One of the most effective strategies for prolonging the lifespan of plastic bioelectronic materials is to prevent damage before it occurs.^[^ ^47^ ^]^ Mechanical stress often leads to the breakdown of covalent bonds, thereby compromising the material's properties and performance.^[^ ^97^ ^]^ Enabling self‐protection by engineering high toughness and anti‐swelling characteristics ensures resistance to both mechanical and moisture degradation.^[^ ^98^, ^99^, ^100^ ^]^ In this context, supramolecular hydrogels with enhanced toughness and tear resistance have been developed using a multi‐solvent‐induced gradient aggregation strategy.^[^ ^101^ ^]^ In this strategy, PVA and alkali lignin (AL) were incorporated into a hydrogel matrix, and the structural and mechanical properties were tuned via high‐temperature annealing in a mixed solvent system made up of ethanol and dimethyl sulfoxide/water (DMSO/H_2_O) solution (**Figure** **4**a). This process induced gradient crystallization of PVA and self‐assembly of AL into a non‐covalent network, thereby enhancing the hydrogel's mechanical performance. The gradient structure formed was attributed to differential solvent evaporation. A phenomenon where the slower evaporation of DMSO/H_2_O facilitated dense internal crystallization, whereas the faster evaporating ethanol produced lower surface crystallization. This disparity resulted in a hierarchical structure characterized by a strong hydrogen‐bonded interior and a weak non‐covalent‐bonded exterior (Figure 4a). This hierarchical architecture efficiently redistributes stress and dissipates energy via reversible AL‐derived non‐covalent bonds, leading to enhanced toughness. In addition, the dense internal crystallization and hydrophobic lignin network hinder water penetration, ensuring the hydrogel's anti‐swelling properties. The developed hydrogels exhibited exceptional mechanical properties, including tensile strength (24.8 MPa), modulus (74.4 MPa), compressive strength (60 MPa), toughness (90 MJ m^−3^), and tear resistance (34 000 J m^−2^), approaching the mechanical properties of biological tissues such as cartilage and tendons (Figure 4b). Furthermore, in vivo implantation studies in a rat model confirmed favorable tissue compatibility, evidenced by enhanced collagen formation (Figure 4c).

Another approach for engineering mechanically robust and anti‐swelling resistance plastic bioelectronic systems involves the development of crack‐resistant anisotropic hydrogels (AHs) integrated with nanofibrils (Figure 4d).^[^ ^102^ ^]^ These were fabricated via “poor solvent evaporation‐assisted hot‐stretching” strategy, which facilitated the formation of aligned multiscale fiber structures within a PVA matrix. The process involved the replacement of DMSO with a glycerol/ethanol mixture, pre‐stretching of the resulting organogel, followed by thermal annealing. During annealing, ethanol evaporation induced tensile stress along the stretch direction, promoting macromolecular chain alignment, crystallization, and nanofibril formation, while glycerol stabilized the structure (Figure 4d). The resulting AHs demonstrated excellent mechanical properties, including tensile strength (33.14 ± 2.05 MPa), toughness (44.1 ± 3.5 MJ m^−3^), and fracture energy (106.18 ± 7.2 kJ m^−2^) (Figure 4e). These properties were attributed to hierarchical structures of the AHs, composed of aligned nanofibrils, crystalline domains, intermolecular hydrogen bonding, and crosslinking networks. This structure enabled efficient crack deflection along the fiber orientations rather than spreading transversely, thereby significantly enhancing self‐protection against crack propagation (Figure 4f). The crack deflection was facilitated by multiple energy dissipation mechanisms such as fiber bundle bridging, nanofibril pullout, and dynamic reconstruction of reversible hydrogen bonds. These multiple energy dissipation mechanisms not only efficiently release energy but also serve as an effective strategy to mitigate mechanical hysteresis under repeated deformation, providing clear advantages for maintaining long‐term mechanical performance. Additionally, anti‐swelling performance was achieved through dense crystalline domains and robust hydrogen bonding, which limited water uptake by promoting polymer chain aggregation. As shown in Figure 4g, the AHs maintained 96% tensile strength and 99% fracture strain retention coefficient after swelling in saline solution for 7 days. In vivo studies revealed that the hydrogel can be implanted into a rat model to replace patellar tendons. The experimental results confirmed that the hydrogels supported collagen regeneration, cell growth, and tissue remodeling, demonstrating their potential to facilitate tendon regeneration (Figure 4h,i).

### 4.2 Autonomous Self‐Healing Polymers for Tissue‐Interfaced Bioelectronics

Despite the remarkable resistance to crack propagation demonstrated by self‐protective plastic bioelectronic materials and systems discussed above, unexpected mechanical damage or accidental cutting may still occur.^[^ ^103^ ^]^ Therefore, the incorporation of self‐healing mechanisms becomes essential for restoring functional performance after damage.^[^ ^104^ ^]^ Autonomous self‐healing can be realized through two primary approaches: extrinsic and intrinsic mechanisms.^[^ ^105^ ^]^ Extrinsic self‐healing mechanism involves introducing healing agents (e.g., microcapsules) that can be released upon mechanical rupture to initiate the healing process.^[^ ^75^, ^106^, ^107^, ^108^, ^109^, ^110^ ^]^ However, such systems are typically limited to one‐time healing events and are incapable of repairing recurrent damage at the same site.^[^ ^111^, ^112^ ^]^ On the contrary, the intrinsic self‐healing mechanism relies on the integration of dynamic reversible bonds within the polymer matrix, enabling multiple cycles of damage and repair without external stimuli.^[^ ^113^, ^114^, ^115^ ^]^ As a result, intrinsic mechanisms are increasingly favored for the development of autonomous self‐healing polymers in bioelectronic applications.

Ionogels, a class of solid‐state electrolytes composed of ionic liquids embedded within a polymer matrix, have emerged as versatile materials for flexible and bioelectronic applications. By combining the high ionic conductivity and non‐volatility of ionic liquids with the stretchability and mechanical compliance of polymers, ionogels offer a unique balance of electrical and mechanical properties. Critically, the multiple physical interactions between ionic liquids and the polymer network–including electrostatic forces, dipole‐dipole interactions, and ion‐dipole interactions–impart intrinsic self‐healing capability. This functionality is particularly advantageous for tissue‐interfaced bioelectronics, where maintaining both electrical conductivity and mechanical integrity under continuous deformation or accidental damage is essential.^[^ ^116^, ^117^ ^]^ Building upon these principles, an intrinsically conductive and self‐healing ionogel was recently developed by integrating long alkyl side chains and ionic liquids into a fluorine‐rich polyacrylate elastomer.^[^ ^118^ ^]^ The long alkyl side chains enabled thermally induced phase transition from semicrystalline to amorphous within temperature ranges of 20–30 °C, which governs the ionogel's mechanical properties, adhesion, and conductivity (Figure 4j). Above the transition temperature, the material exhibited strong adhesion to skin, whereas below this threshold, it stiffened and detached easily, ensuring painless and injury‐free removal. Self‐healing was achieved via dipole‐dipole and ion‐dipole interactions between the fluorinated polymer chains and the ionic components. Upon damage, the ionogel exhibited a mechanical self‐healing efficiency of up to 80% and 52.5% of its mechanical properties under ambient and underwater conditions, respectively, after 24 h (Figure 4k). Here, the mechanical self‐healing efficiency of the sample is defined as the ratio of [recovered strain of healed sample] / [strain of original sample]. In contrast to the slow recovery of mechanical properties, which relies on the rearrangement and re‐entanglement of polymer chains over extended timescales, the electrical performance of this material can be restored within ≈10 s, as the recovery of electrical performance involves the rapid reconnection of disrupted conductive pathways.^[^ ^119^, ^120^ ^]^ It was further employed as a bioelectrode for real‐time acquisition of Electrocardiogram (ECG) and EMG signals underwater, which outperformed commercial Ag/AgCl electrodes in signal fidelity (Figure 4l). However, the material's relatively slow healing rate (24 h) does not meet the 1 min recovery threshold required for commercial applications.^[^ ^121^, ^122^ ^]^

To address this limitation, a rapidly self‐healing electronic skin (e‐skin) has been designed for real‐time electrophysiological and motion monitoring.^[^ ^122^ ^]^ This e‐skin was constructed using a thermoplastic polyurethane (TPU) matrix incorporating isophorone diisocyanate (IPDI) and dynamic disulfide bonds as functional moieties (Figure 4m). The dynamic disulfide bonds facilitated autonomous, reversible bond formation through thiol‐disulfide metathesis reactions, enabling rapid self‐healing within 10 s without external stimuli (Figure 4n). The asymmetric alicyclic structure of IPDI enhances polymer chain mobility, thereby accelerating the disulfide exchange reactions even at ambient temperatures. This system achieved a mechanical self‐healing efficiency of 80% in ambient and underwater conditions. Here, the mechanical self‐healing efficiency of the sample is defined as the ratio of [recovered modulus of healed sample] / [modulus of original sample]. Furthermore, the e‐skin successfully enabled noninvasive, real‐time monitoring of ECG and EMG signals even after damage or underwater exposure and demonstrated sensitivity to subtle physiological motions (including blinking, speech, respiration, joint movements) and cardiac activity (Figure 4o). Thus, fast self‐healing mechanisms are critical for long‐term tissue‐interfaced biomedical applications, enabling devices to maintain stable functionality even after unexpected mechanical damage, ensuring operational reliability and extended device lifespan.

### 4.3 Autonomous Biodegradable Polymers for Tissue‐Interfaced Bioelectronics

**Figure 5:**
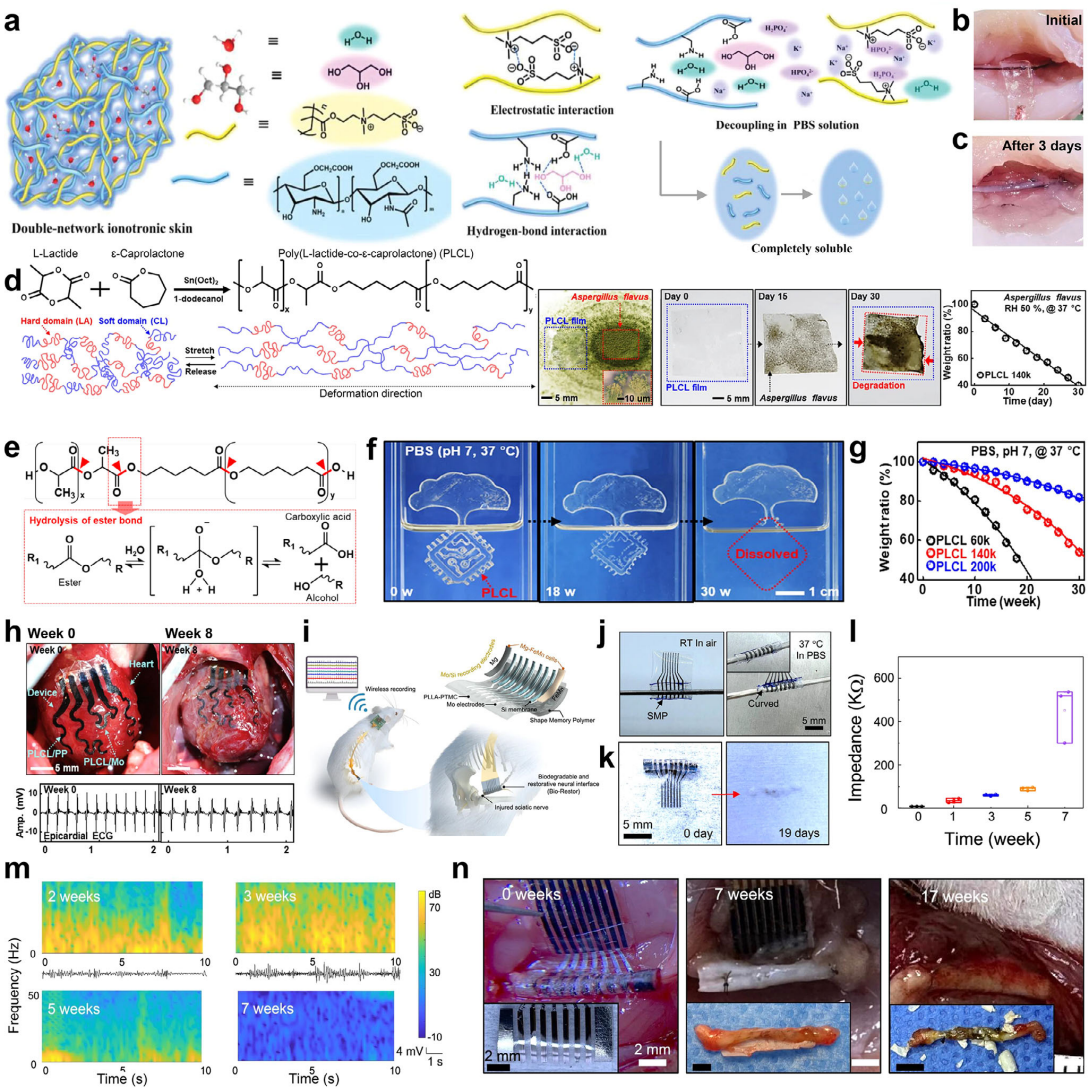
Autonomous degradable polymers for tissue‐interfaced bioelectronics. a) Double‐network structure of ionotronic skin composed of hydrogen‐bonded CCS and electrostatically cross‐linked SBMA. b,c) Demonstration of SBMA_40_‐CCS_1_‐Gly_2_ as biodegradable electrodes for action potential recording and neural stimulation, showing progressive degradation on the sciatic nerve from the initial state to day 3. a–c) Reproduced with permission.^[^ ^128^ ^]^ Copyright 2023, Wiley‐VCH. d) Chemical structure of the PLCL elastomer derived from the ring‐opening copolymerization of biocompatible and biodegradable monomers L‐lactide and ε‐caprolactone. e) Hydrolytic degradation mechanism of PLCL involving ester bond cleavage, producing biocompatible lactic and caproic acids. f) Optical images showing dissolution stages of a 100 µm‐thick PLCL (140k) film in PBS (pH 7) at 37 °C. g) Time‐dependent weight ratio changes of PLCL films (100 µm thick) as a function of molecular weight. h) Photographs of implanted cardiac jackets (top) and representative epicardial ECG signals recorded by the devices (bottom) at weeks 0 and 8 after in vivo implantation. d–h) Reproduced with permission.^[^ ^34^ ^]^ Copyright 2023, Springer Nature. i) Schematics of the Bio‐Restor neural interface system. j) Photographs of the neural interface in its transient flat form under ambient air at room temperature (left), and in its final rolled‐up configuration after immersion in PBS (pH 7.4, 37 °C) (right). k) Sequential images showing the accelerated dissolution of Bio‐Restor in PBS (pH 7.4, 60 °C) over a 19‐day period. l) Impedance measurements for Mo/Si bioresorbable electrodes before and after 7 weeks in PBS. m) Representative signal traces and time‐frequency spectrograms at various biodegradation stages. n) Images of Bio‐Restor implanted at sciatic nerve injury sites in rodents over a 17‐week degradation period. i–n) Reproduced with permission.^[^ ^130^ ^]^ Copyright 2025, Springer Nature.

While self‐healing is a critical feature for restoring functional properties upon mechanical damage, repeated damage healing cycles can ultimately lead to molecular fatigue or reduced dynamic bonding efficiency.^[^ ^123^ ^]^ Over time, even highly robust self‐healing systems may experience a decline in healing efficiency, electrical property, and the ability to fully recover original functionality.^[^ ^124^ ^]^ Furthermore, plastic bioelectronic devices designed for temporary therapeutic or diagnostic purposes may become obsolete at the end of their service life. In the case of implantable devices, their prolonged presence can trigger chronic inflammation or immune responses, and also, improper disposal can contribute to electronic waste (e‐waste).^[^ ^125^, ^126^, ^127^ ^]^ To address these concerns, self‐degradation becomes essential for enabling transient functionality, followed by safe and autonomous disintegration after use. In this context, a fully biodegradable and biocompatible ionotronic skin was developed for transient bioelectronic applications.^[^ ^128^ ^]^ This ionotronic skin was fabricated using a double‐network strategy based on natural polyelectrolyte derivatives comprising carboxylated chitosan (CCS) and polymerized sulfobetaine methacrylate (SBMA) (**Figure** **5**a). The degradation mechanism relies on the water solubility of CCS and the dissociation of SBMA‐based electrostatic networks. Notably, the SBMA polymers exhibited complete disintegration in phosphate‐buffered saline (PBS) at room temperature within 3 days. This rapid degradation was attributed to the solubility of CCS and the low dissociation energy (6.1 kJ mol^−1^) of SBMA dimers. In practical applications, the ionotronic skin was employed as a transient bioelectronic interface for monitoring various electrophysiological signals, including ECG, EMG, electrooculogram, and electroencephalogram. Additionally, the device was implanted on a bullfrog's sciatic nerve, enabling both compound action potential recording and neural stimulation (Figure 5b,c). The ionotronic skin fully disintegrated in vivo within 3 days, with the corresponding loss of electrical signal, eliminating the need for surgical removal.

However, the rapid disintegration rate (within 3 days) limits its application in long‐term therapeutic and monitoring situations.^[^ ^129^ ^]^ To overcome this limitation, ultra‐stretchable and biodegradable elastomers based on poly(l‐lactide‐co‐ε‐caprolactone) (PLCL) were developed for soft transient electronics (Figure 5d).^[^ ^34^ ^]^ These elastomers exhibited exceptional mechanical properties, including stretchability up to 1600%, crack resistance, and programmable biodegradability. The programmable degradation mechanism of PLCL was governed by hydrolytic cleavage of ester bonds, enabling gradual degradation over 30 weeks in a PBS (Figure 5e,g). The PLCL elastomer was utilized to fabricate a suture‐free cardiac jacket integrated with conductive composites and implanted onto rat hearts (Figure 5h). This device supported epicardial ECG monitoring, electrical stimulation, and myocardial strain sensing. In vivo, the device demonstrated controlled degradation over 8 weeks, with minimal physical disintegration due to an encapsulation layer that delayed water ingress. In another design strategy, a fully biodegradable and restorative neural interface (Bio‐Restor) was engineered for real‐time monitoring and therapeutic intervention in long‐gap peripheral nerve injuries.^[^ ^130^ ^]^ The Bio‐Restor system consisted of a 7‐channel molybdenum/monocrystalline silicon (Mo/Si) electrode array for neural recording, a galvanic cell composed of magnesium and iron manganese (Mg/FeMn) alloy for electrical stimulation to promote nerve regeneration, and a shape‐memory substrate of poly(l‐lactic acid) and poly(trimethylene carbonate) (PLLA‐PTMC) for self‐morphing behavior at physiological temperatures (Figure 5i). In situ experiments confirmed the self‐rolling behavior of the device, allowing it to wrap securely around the sciatic nerve to record neural signals (Figure 5j). The constituent components degraded sequentially both in PBS and in vivo. At physiological temperature (37 °C), dissolution was slow, ≈5.5–8.5 weeks (Figure 5j), whereas accelerated degradation at 60 °C in PBS completed within 20 days (Figure 5k). The Si layer acted as a water barrier, extending the recorded functionality for ≈5.5–8.5 weeks at 37 °C (Figure 5l). Therefore, the slow degradation under physiological conditions ensures long‐term operational stability of the device, while the accelerated degradation at elevated temperatures provides an effective strategy for device disposal after functional lifetime. This tunable degradation behavior offers the advantage of precisely controlling the device lifespan according to application requirements. Implanted on the sciatic nerve, the Bio‐Restor device enabled simultaneous electrical stimulation and neural signal acquisition at long‐gap nerve injury sites in rats. The integrated galvanic cell promoted axonal regeneration, while the Mo/Si electrode array continuously monitored regeneration progress. In vivo, the Mo/Si electrodes lost recording capability by week 7 (Figure 5m), and the PLLA‐PTMC substrate underwent gradual hydrolysis, completing degradation over several weeks (Figure 5n). This controlled degradation eliminated the need for surgical removal, thereby minimizing the risk of chronic immune responses.

## 5 Self‐Sensing Polymers for Tissue‐Interfaced Bioelectronics

Sensing is a fundamental aspect of tissue‐interfaced bioelectronic systems, enabling continuous monitoring of physiological conditions and dynamic biological signals for real‐time health assessment, early diagnosis, and therapeutic feedback. Conventional bioelectronic platforms, however, often encounter challenges in capturing such dynamic cues with sufficient fidelity under long‐term or ambulatory conditions, which underscores the need for sensing materials that can seamlessly integrate with soft and heterogeneous tissue environments. Self‐sensing polymers represent a promising solution in this context. Their inherent softness, adaptability, and tunable chemical structures allow intimate integration with tissues, while their ability to autonomously transduce endogenous stimuli‐mechanical, thermal, and biochemical (e.g., pH)‐into measurable signals provides a reliable foundation for autonomous monitoring without reliance on bulky external instrumentation. By directly coupling polymer responsiveness to physiological events, these systems enable tissue‐conformal sensing that is both minimally invasive and clinically relevant. This section categorizes self‐sensing polymers based on their responsiveness to these distinct physiological signals and highlights recent advances that leverage such stimuli for autonomous monitoring and feedback control.

### 5.1 Autonomous Mechanical‐Responsive Polymers

**Figure 6:**
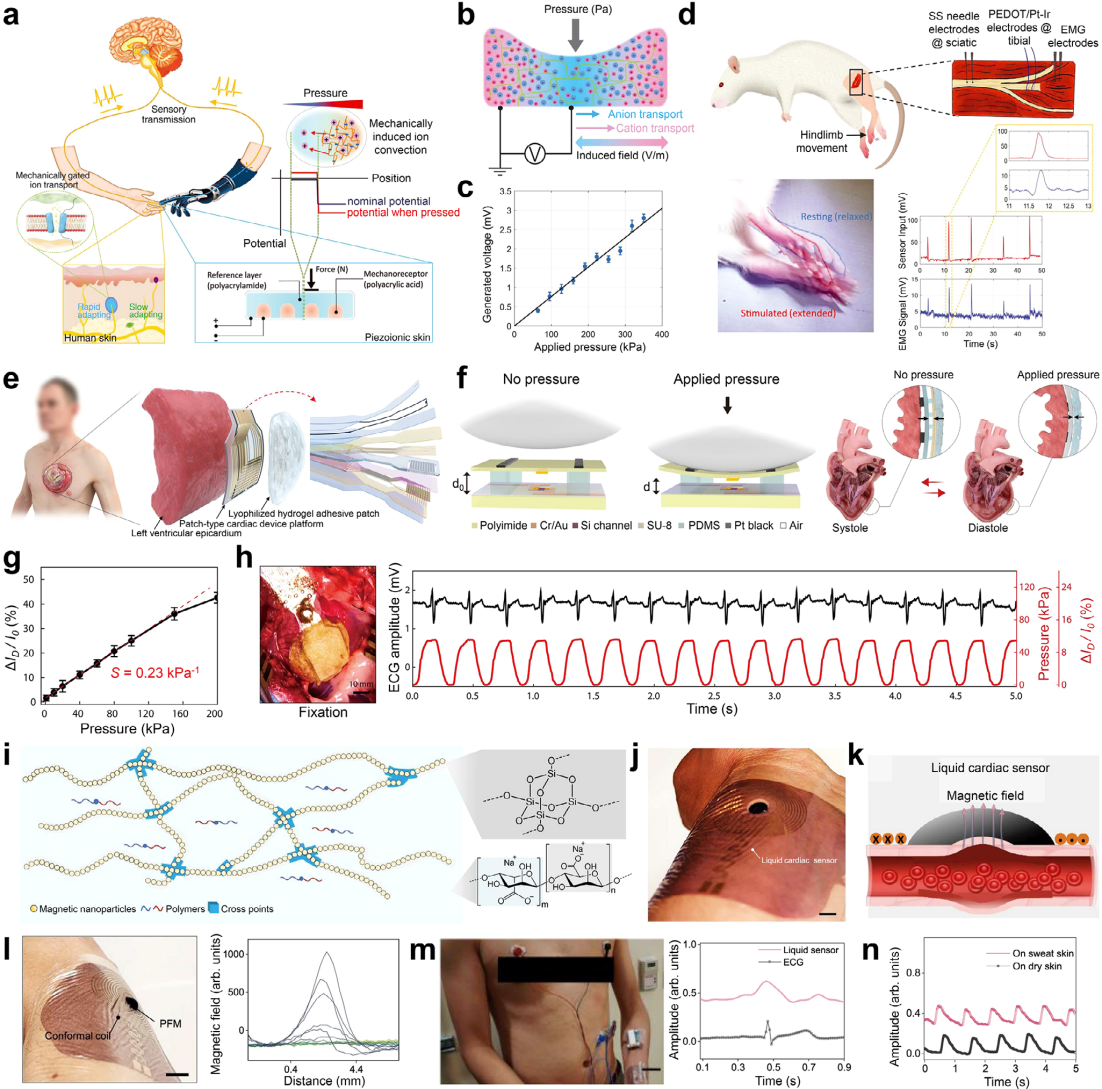
Autonomous mechano‐responsive polymers for tissue‐interfaced bioelectronics. a) Schematics of biological sensory transduction and the piezoionic sensing device functioning as an iontronic neuroprosthetic. b) Illustration of the sensing mechanism of the piezoionic sensor under applied pressure. c) Peak output voltage versus applied pressure. d) Setup of the piezoionic nerve stimulation experiment. a–d) Reproduced with permission.^[^ ^137^ ^]^ Copyright 2022, American Association for the Advancement of Science. e) Schematic of the device integrating pressure‐sensitive transistors and pacing electrodes attached to the epicardium via a hydrogel patch for cardiac monitoring and stimulation. f) Schematic representation of an air‐dielectric transistor used for compressive pressure sensing, illustrating its sensing mechanism during cardiac contraction (systole) and relaxation (diastole). g) Variation in drain current in response to applied compressive pressure. h) Patch attached to the left ventricular epicardium via Ag–CA–Ca^2^⁺ adhesive. Pressure signal (red) from the transistor compared with surface ECG (black). e–h) Reproduced with permission.^[^ ^138^ ^]^ Copyright 2022, American Association for the Advancement of Science. i) Diagram showing nanoparticle chain alignment in the PFM, including carrier fluid structures composed of silicon dioxide and sodium alginate. j) Image of the liquid‐based cardiac sensor. k) Schematic illustrating how the liquid cardiac sensor droplet conforms to complex surface topography. l) Image of the liquid cardiac sensor on the wrist and the magnetic field of a single droplet. m) Experimental setup for simultaneous measurement of the liquid sensor and ECG, showing pulse wave characteristics captured by the sensor. n) Evaluation of liquid cardiac sensors on both dry and sweaty skin. i–n) Reproduced with permission.^[^ ^140^ ^]^ Copyright 2024, Springer Nature.

In recent decades, advances in mechanically responsive devices (e.g., pressure and strain sensitive systems) have enabled the development of innovative invasive and wearable technologies capable of accurately monitoring physiological signals in clinical diagnosis and therapeutic treatments in tissue‐interfaced applications.^[^ ^131^, ^132^, ^133^, ^134^, ^135^, ^136^ ^]^ As shown in **Figure** **6**a, inspired by biological mechanoreceptors, a hydrogel‐based piezoionic sensor has been developed to transduce mechanical deformations into ionic currents for neuromodulation.^[^ ^137^ ^]^ The hydrogel detected pressure via deformation‐driven ion flux, where mechanical compression induced differential transport of mobile ions, leading to charge separation and voltage generation (Figure 6b). This voltage generation resulted from streaming potentials produced by convective ion flow within the hydrogel matrix. The device exhibited a pressure sensitivity 8 µV kPa^−1^ (Figure 6c). The piezoionic mechanoreceptor hydrogel was employed as a self‐powered tactile sensor for peripheral nerve stimulation and EMG recording in rodents (Figure 6d). Under pressure, the device generated ionic currents that were delivered to the sciatic or tibial nerves via stainless steel needle or PEDOT‐coated electrodes, successfully inducing compound action potentials. EMG signals recorded from the corresponding muscles confirmed neuromodulation. However, during prolonged operation under dry conditions, signal drift was observed over repeated cycles, limiting long‐term reliability in wearable or implantable applications.

To ensure stable long‐term performance in both dry and wet conditions, an array of pressure‐sensitive air‐dielectric transistors has been developed for simultaneous mechanophysiological sensing and therapeutic electrical stimulation (Figure 6e).^[^ ^138^ ^]^ This system included a 10 × 10 active‐matrix transistor array for real‐time epicardial pressure mapping, platinum black‐coated pacing electrodes for efficient low‐threshold electrical stimulation, and a catechol‐functionalized alginate‐based hydrogel for secure adhesion to wet epicardial surfaces. The sensing mechanism relied on pressure‐induced modulation of capacitance between the gate and channel (Figure 6f). Under pressure, or during cardiac relaxation (diastole), the expanding left ventricle exerted compressive pressure on the device, deforming the elastomeric top gate and reducing the air‐gap dielectric thickness. This increased the capacitance, resulting in enhanced drain current. On the other hand, during contraction (systole), reduced pressure restored the air gap, leading to a decrease in drain current. The device achieved a sensitivity of 0.23 kPa^−1^ (Figure 6g). Notably, it was employed to monitor epicardial pressure in synchronization with ECG signals in a live rabbit model (Figure 6h). Additionally, the system enabled electrical pacing and defibrillation to restore intrinsic heart rhythms while continuously monitoring cardiac beating motions without signal interference from electric simulation. Despite its excellent long‐term reliability, the device utilized an external alginate‐based adhesive, which is prone to swelling upon fluid absorption.^[^ ^139^ ^]^ This swelling can potentially affect pressure transmission fidelity and compromise signal accuracy over time.

To address this challenge, a reconfigurable and conformable liquid cardiac sensor based on a permanent fluidic magnet (PFM) was developed for continuous ambulatory cardiac monitoring.^[^ ^140^ ^]^ The PFM consisted of a 3D oriented and ramified magnetic network of non‐Brownian ferromagnetic nanoparticles suspended in a flowable carrier fluid (Figure 6i). When applied to the skin (Figure 6j), the PFM conformed seamlessly to skin features such as wrinkles and creases, maintaining intimate contact during motion without requiring external pressure or adhesives. The sensing mechanism relied on magnetic flux variations induced by arterial pulse waves. As blood pulses through the underlying arteries, biomechanical displacement alters the volume distribution of the PFM, modulating the local magnetic field (Figure 6k). These flux variations were detected by a conformal soft coil, which converted magnetic changes into electrical signals via electromagnetic induction (Figure 6l). The amplitude and frequency of the induced signals correlated with pulse wave characteristics, enabling real‐time monitoring of heart beating, blood pressure, and arterial wall stiffness (Figure 6m). The PFM sensor maintained performance under mechanical deformation, sweating, and stretching, offering high‐fidelity cardiovascular monitoring even in ambulatory settings with minimal interference (Figure 6n).

### 5.2 Autonomous Thermal‐Responsive Polymers

**Figure 7:**
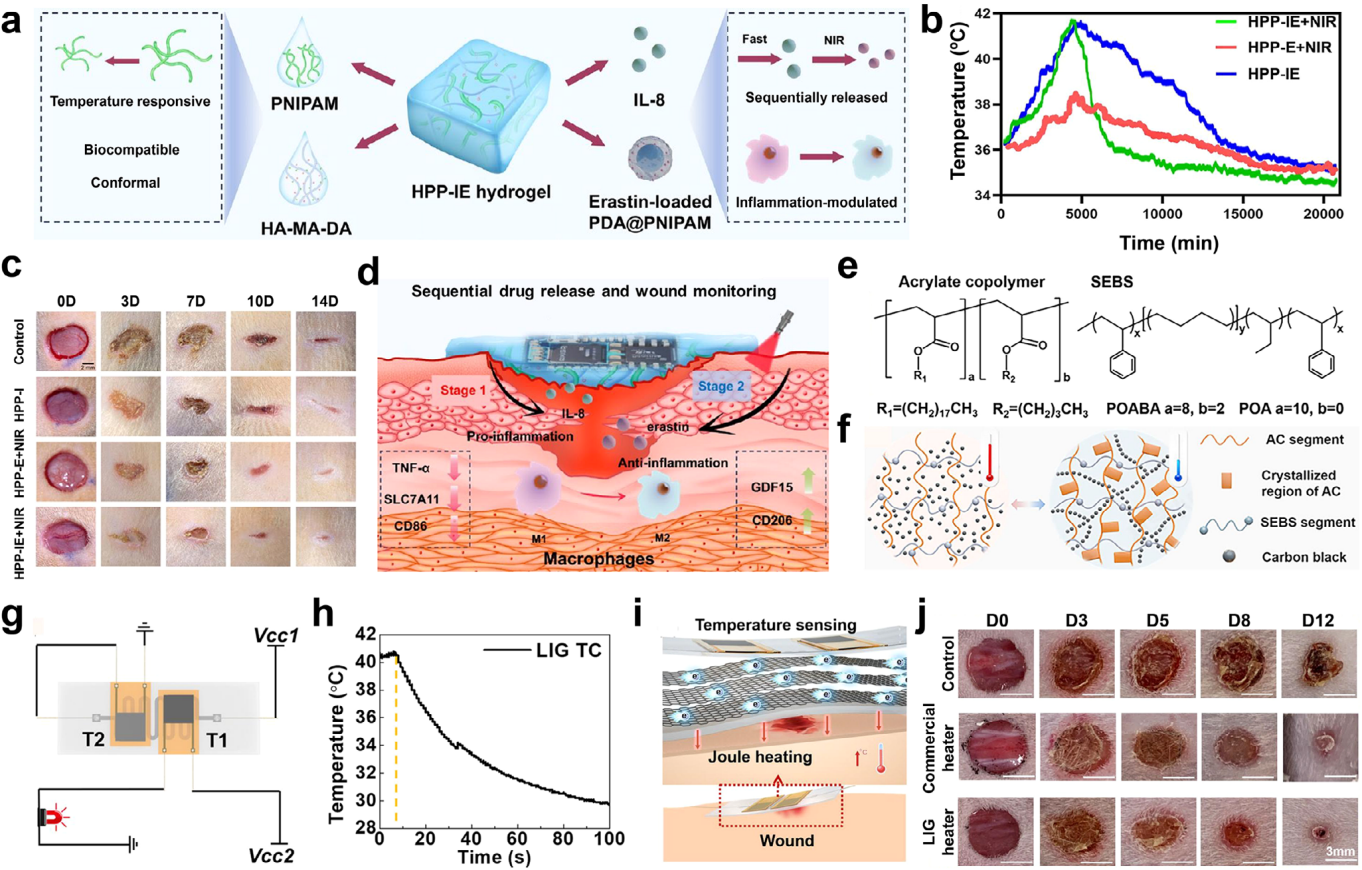
Autonomous thermal‐responsive polymers for tissue‐interfaced bioelectronics. a) Schematic illustration of an HPP‐IE hydrogel composed of HA‐MA‐DA and PNIPAM, exhibiting temperature‐responsive drug delivery of IL‐8 and erastin. b) In vivo wound temperature profiles under various treatment conditions, demonstrating inflammation‐associated thermal fluctuations. c) Photographic images showing the wound healing progression over 14 days under different treatment conditions in mouse models. d) Sequential drug release modulated by body temperature and inflammatory microenvironment. a–d) Reproduced with permission.^[^ ^146^ ^]^ Copyright 2024, American Chemical Society. e) Molecular structures of acrylate copolymer and SEBS used in the thermally switchable conductive matrix. f) Schematic illustration of the temperature‐induced crystallization and conductivity switching behavior of the AC/SEBS composite. g) Circuit design enabling feedback‐controlled protection and regulation in thermal interface systems. h) Real‐time thermal response of a LIG‐based temperature controller. i) Conceptual illustration of a thermal‐responsive electronic system interacting with wound tissue. j) Photographs showing wound healing efficacy in comparison with control devices. e–j) Reproduced with permission.^[^ ^147^ ^]^ Copyright 2024, Springer Nature.

Temperature is a fundamental physiological parameter that fluctuates in response to various biological processes, including inflammation, infection, and wound healing.^[^ ^141^ ^]^ These endogenous thermal variations provide natural and noninvasive signals for autonomous modulation of tissue‐interfaced bioelectronic systems.^[^ ^142^, ^143^, ^144^ ^]^ Unlike externally applied stimuli, temperature changes can be harnessed to enable self‐regulating therapeutic activation. However, the design of responsive materials that can precisely operate within the narrow physiological temperature range remains a significant challenge.^[^ ^145^ ^]^ To address this challenge, a temperature‐responsive hydrogel dressing (termed HPP‐IE) with a two‐stage drug release process was developed.^[^ ^146^ ^]^ This system combined poly(N‐isopropylacrylamide) (PNIPAM), a well‐established lower critical solution temperature polymer, with hyaluronic acid modified with methacrylate and dopamine (HA‐MA‐DA). PNIPAM underwent a reversible sol‐gel phase transition near 32 °C, while HA‐MA‐DA enhanced biocompatibility and enabled strong tissue adhesion through catechol‐mediated bonding (**Figure** **7**a). The HPP‐IE was engineered to leverage inflammation‐induced hyperthermia for temporally controlled sequential drug delivery. Macrophages can polarize toward either pro‐inflammatory M1 or anti‐inflammatory, pro‐regenerative M2 phenotypes, with the latter being essential for inflammation resolution and tissue repair. Upon moderate temperature elevations (≈38 °C), the hydrogel released interleukin‐8, promoting immune cell recruitment. At elevated temperatures (> 40 °C), erastin was subsequently released to induce M2 macrophage polarization and mediate anti‐inflammatory responses. Thermal profiles of inflamed tissue confirmed that this dual‐release mechanism was autonomously regulated by endogenous temperature fluctuations (Figure 7b). In murine wound models, application of the HPP‐IE hydrogel accelerated epithelial regeneration and reduced fibrotic tissue formation (Figure 7c). It functioned as a temperature‐sensitive biomaterial that exerted stage‐specific therapeutic actions in response to sequential thermal signs (Figure 7d). Despite its promising biological performance, this system operated through passive mechanisms (thermal diffusion and polymer swelling), which limits its spatiotemporal precision and preclude active feedback control.

To overcome these constraints, an advanced material‐electronic integration strategy was introduced using a thermally switchable conductive polymer matrix. This composite was fabricated by blending a styrene‐ethylene‐butylene‐styrene (SEBS) elastomer with a thermosensitive acrylate copolymer (AC), which exhibited reversible conductivity switching across defined temperature thresholds (Figure 7e).^[^ ^147^ ^]^ In this design, SEBS served as a mechanically flexible and durable matrix, while the AC component functioned as the thermally active segment. At lower temperatures, the AC domains formed partially crystalline structures that disrupted the percolation pathways of conductive fillers (e.g., carbon black), resulting in reduced electrical conductivity. Upon heating, the crystalline regions melted, increasing polymer chain mobility and re‐establishing conductive networks. This transition enabled reversible, temperature‐mediated electrical modulation (Figure 7f). To enable real‐time thermal sensing, the thermoresponsive matrix was integrated with a laser‐induced graphene (LIG)‐based thermistor featuring a dual transistor architecture (Figure 7g). Owing to its porous morphology and high thermal responsiveness, the LIG thermistor rapidly detected subtle temperature changes with high sensitivity (Figure 7h). When embedded within a self‐regulating feedback system, the thermistor could autonomously trigger Joule heating via an integrated heater in response to inflammation‐induced thermal shifts. This capability enabled localized thermal therapy in a self‐regulating manner, resulting in the formation of a temperature‐responsive patch (Figure 7i). In vivo application of this system in murine wound models, demonstrated significantly improved therapeutic outcomes compared to both untreated controls and conventional heating devices. The LIG‐integrated patch accelerated wound closure, reduced inflammation, and promoted tissue regeneration. These outcomes were attributed to the patch's ability to deliver precise dynamic thermal stimulation synchronized with the physiological wound environment (Figure 7j). This approach illustrates the potential of integrating thermally active polymer systems with real‐time electronic control units to regulate adaptive therapeutic systems for responsive bioelectronic applications.

### 5.3 Self‐Regulated pH‐Responsive Polymers

pH, alongside temperature, serves as a critical physiological indicator that dynamically reflects the biochemical status of biological tissues. Deviations from normal pH levels are closely associated with pathological conditions such as inflammations, bacterial infections, ischemia, and chronic wounds.^[^ ^148^ ^]^ In particular, alkaline microenvironments often signal bacterial proliferation or prolonged inflammatory responses, whereas acidic shifts (acidosis)^[^ ^149^, ^150^ ^]^ can occur during bacterial metabolism, ischemic injury, or tumor progression. A return toward neutral pH typically indicates tissue recovery.^[^ ^151^ ^]^ Accordingly, the development of pH‐responsive polymer systems capable of autonomously sensing and adapting to these fluctuations is vital for next‐generation tissue‐interfaced bioelectronic systems.

**Figure 8:**
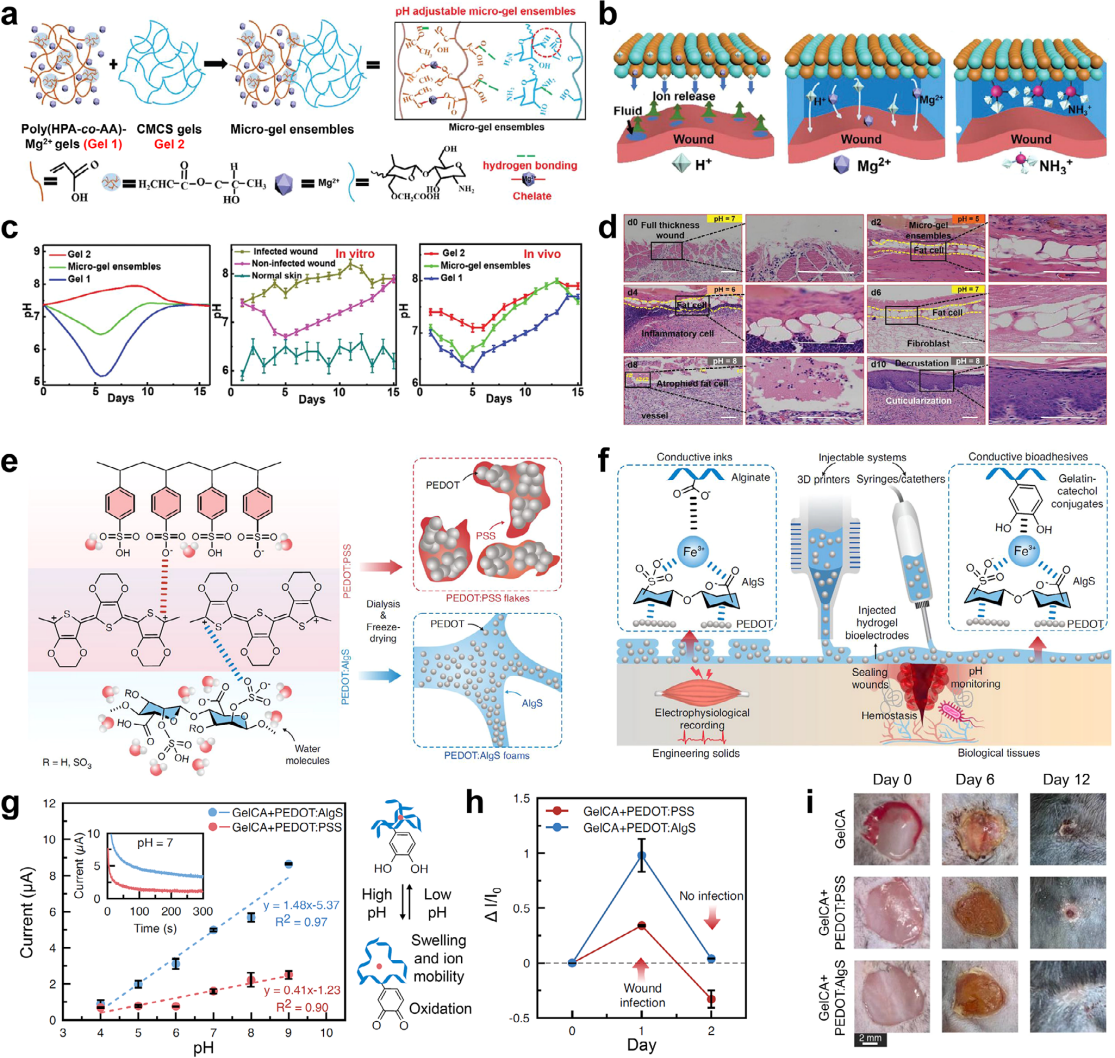
Self‐regulated pH‐responsive polymers for tissue‐interfaced bioelectronics. a) Synthetic route of a self‐assembling micro‐gel ensemble via hydrogen bonding between Gel 1 and Gel 2. b) Autonomous pH‐regulating mechanism of the micro‐gel ensemble. c) Integrated analysis of in vitro and in vivo pH dynamics across different gel formulations and wound types. d) Histological and fluorescence staining of regenerated tissue following ensemble treatment in wound models. a–d) Reproduced with permission.^[^ ^152^ ^]^ Copyright 2022, Wiley‐VCH. e) Molecular design of PEDOT‐based conductive polymers doped with sulfonated macromolecules (AlgS and PSS). f) Injectable and 3D‐printable PEDOT:AlgS hydrogels enabling wearable bioelectronics for continuous wound monitoring and closure. g) pH sensitivity of conductive bioadhesives evaluated via chronoamperometry across multiple pH levels. h,i) In vivo monitoring of infection progression and wound healing using pH‐responsive, self‐regulated bioelectronic hydrogels with integrated sensing and therapeutic functionality. e–i) Reproduced with permission.^[^ ^153^ ^]^ Copyright 2025, Springer Nature.

To address this need, a self‐assembling pH‐responsive hydrogel ensemble was engineered by combining two distinct gel components: Gel 1 was composed of poly(HPA‐co‐AA)‐Mg^2+^, and Gel 2 consisted of carboxymethyl chitosan (CMCS) coordinated with Cd^2+^ ions. Together, these two components formed a supramolecular hydrogel network via reversible hydrogen bonding interactions. This architecture provided autonomous buffering functionality, gradually releasing ions such as NH_3_^+^ and Mg^2+^ to dynamically regulate the local wound environment (**Figure** **8**a).^[^ ^152^ ^]^ This microgel ensemble exhibited real‐time responsiveness to biochemical changes, maintaining the wound pH within a therapeutically optimal range (Figure 8b). Both in vitro and in vivo studies validated its ability to preserve pH homeostasis across various wound types, including infected and non‐infected models (Figure 8c). Furthermore, histological and fluorescence analyses confirmed that the treatment reduced inflammatory cell infiltration and enhanced collagen deposition, indicating effective tissue regeneration mediated by autonomous pH modulation (Figure 8d).

Despite these advantages, the hydrogel's chemical buffering strategy lacks intrinsic sensing capability and offers only limited spatiotemporal control. It passively stabilizes the wound environment but does not actively monitor or respond to evolving biochemical signals. To overcome these limitations, a second strategy was implemented through the development of a pH‐sensitive conductive polymer composite for real‐time monitoring and electrical feedback.^[^ ^153^ ^]^ This system employed PEDOT doped with PSS and alginate sulfate (AlgS) to yield PEDOT:PSS and PEDOT:AlgS hydrogel composites, respectively (Figure 8e). Among these, the PEDOT:AlgS hydrogel composite exhibited superior properties, including a 35‐fold increase in conductivity due to the high dispersion of AlgS dopant, excellent hydration capacity, and a 250% improvement in pH sensitivity, making it highly suitable for integration into pH‐responsive bioelectronic systems. Designed as an injectable and ionically crosslinked hydrogel, the PEDOT:AlgS composite also demonstrated strong tissue adhesion and hemostatic capability with a burst pressure more than five times higher than that of commercial alternatives. These properties enabled robust real‐time pH monitoring in dynamic tissue environments (Figure 8f). Chronoamperometric measurements confirmed a linear and reproducible current response across a biologically relevant pH range (4–10), encompassing both inflammatory and healing phases (Figure 8g). In vivo experiments further validated the hydrogel's utility in early infection detection and tracking wound healing progress. Notably, electrical impedance changes were found to correlate precisely with different stages of inflammation, enabling timely modulation of therapeutic interventions. Compared to conventional hydrogels and PEDOT:PSS‐only hydrogels, the PEDOT:AlgS‐based hydrogel exhibited superior wound closure and tissue regeneration, as confirmed through both visual inspection and histological evaluation (Figure 8h,i). In addition to wound monitoring, the PEDOT:AlgS hydrogel also supported electrophysiological signal acquisition including ECG and EMG, highlighting its broader potential for bioelectronic applications beyond wound healing.

## 6 Autonomous Multi‐Frameworks‐Integrated Polymers for Tissue‐Interfaced Bioelectronics

**Figure 9:**
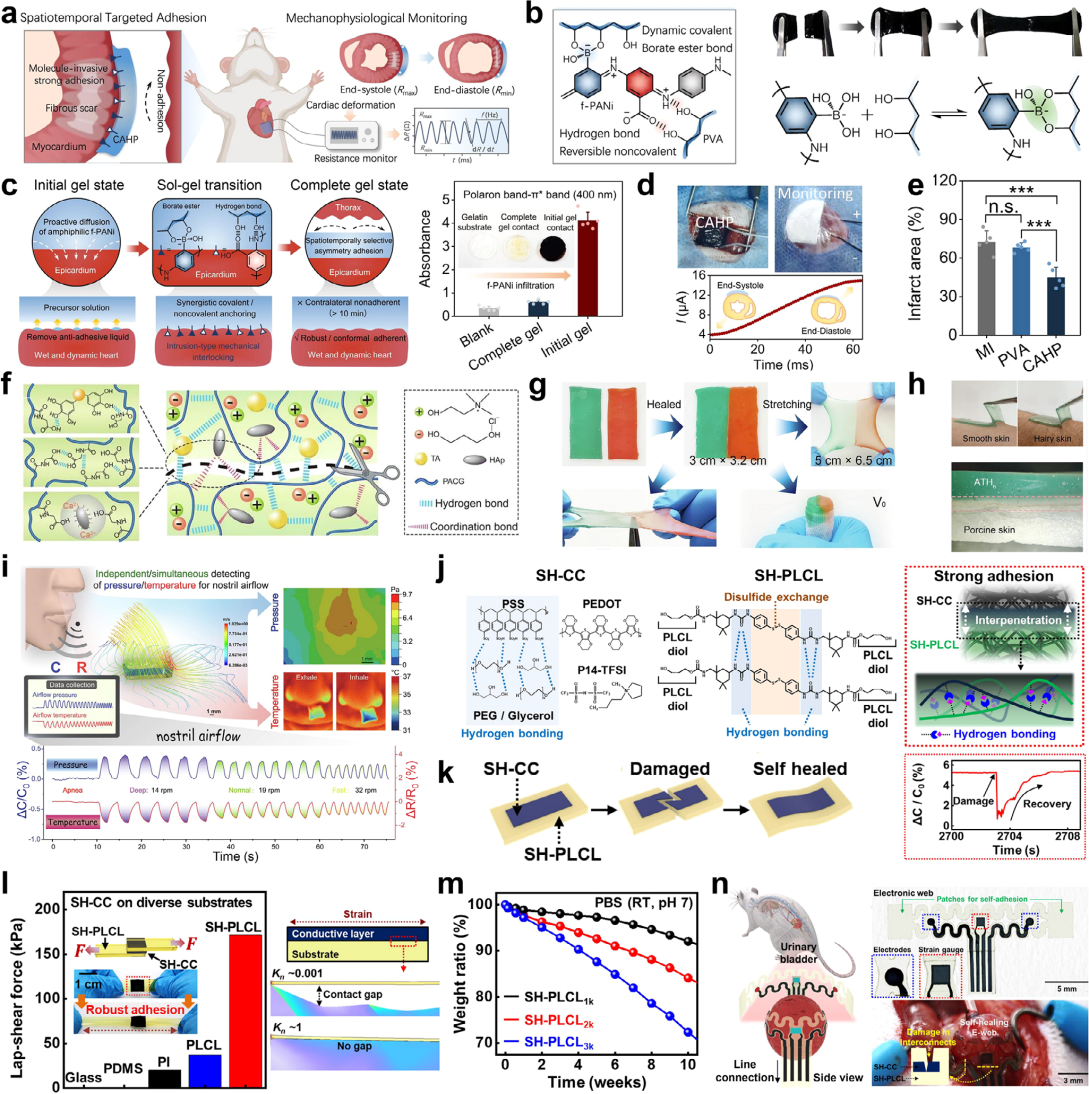
Autonomous multi‐framework‐integrated polymers for tissue‐interfaced bioelectronics. a) Illustration of a CAHP enabling synchronized cardiac mechanical sensing and electrocoupling‐based therapy. b) Dynamic covalent borate ester bonds and non‐covalent hydrogen bonds at the interface between functionalized f‐PANi and PVA. c) Temporal evolution of the adhesion mechanism of CAHP, along with corresponding absorbance changes at 400 nm during initial and complete gel states. d) Photographs of CAHP mounted on the left ventricular surface for mechanophysiological monitoring, with corresponding current changes recorded from systole to diastole. e) Quantification of infarct size in MI, PVA, and CAHP groups. a–e) Reproduced with permission.^[^ ^156^ ^]^ Copyright 2023, Springer Nature. f) Self‐healing mechanism of ATH6 eutectogel. g) Demonstration of ATH6 eutectogel's resistance to extreme mechanical stimuli and h) its adhesion to smooth skin, hairy skin, and porcine tissue, showing seamless contact. i) Simultaneous detection of nostril airflow pressure and temperature via distinct capacitance and resistance signals. f–i) Reproduced with permission.^[^ ^158^ ^]^ Copyright 2025, Springer Nature. j) Material design chemistry of SH‐PLCL and SH‐CC. k) Self‐healing process of SH‐CC on SH‐PLCL. l) Robust SH‐CC adhesion between SH‐PLCL layers before and after stretching, with lap shear comparison across substrates. m) Time‐dependent weight change of SH‐PLCL films with different Mn values in PBS (pH 7, RT). n) Self‐healing e‐web integrated onto a rat bladder via self‐healing patches for monitoring and regulation of biophysiological functions. j–n) Reproduced with permission.^[^ ^33^ ^]^ Copyright 2024, American Association for the Advancement of Science.

Integrating multiple auto‐POFs into a single bioelectronic device significantly enhances functionality, robustness, and adaptability within dynamic biological environments.^[^ ^154^, ^155^ ^]^ This multifunctionality offers superior advantages over devices incorporating only a single autonomous feature. By synergizing multi‐frameworks such as self‐adhesion, self‐healing, self‐sensing, and self‐degradation, these systems can ensure conformal tissue interfacing and controlled disappearance of the device upon reaching an irreparable state or after fulfilling its function.^[^ ^96^ ^]^ Consequently, diverse autonomous multi‐framework‐integrated systems for tissue‐interfaced applications have been reported. As illustrated in **Figure** **9**a, a chronological adhesive cardiac patch (CAHP) with integrated selective self‐adhesive and self‐healing functionalities was developed for synchronous mechanophysiological monitoring and electrocoupling therapy of myocardial infraction.^[^ ^156^ ^]^ The CAHP was fabricated using functionalized polyaniline (f‐PANi) integrated with polyvinyl alcohol (PVA) to form a multifunctional hydrogel. The f‐PANi formed dynamic covalent borate ester bonds and noncovalent hydrogen bonds with PVA, enabling electroactivity, selective tissue adhesive, and self‐healing (Figure 9b,c). Selective adhesive to myocardium was achieved through a chronological adhesion mechanism enabled by sol‐gel transition. In the initial gel phase, amphiphilic f‐PANi absorbed anti‐adhesive pericardial fluid and penetrated the wet epicardium, forming dynamic borate ester bonds with PVA and creating interlocking structures, resulting in enhanced adhesion to myocardial tissue. Upon complete gelation, the dense crosslinked network limited further polymer diffusion, preventing adhesion to non‐target tissues (Figure 9b). The reversible dynamic borate ester and hydrogen bonding between f‐PANi and PVA facilitated autonomous self‐healing of the hydrogel without external stimuli (Figure 9c). The CAHP was conformally applied to the left ventricle of a live rat model to continuously monitor cardiac mechanophysiology and concurrent electrocoupling therapy (Figure 9d). During systolic and diastolic deformation, volumetric changes in the hydrogel resulted in resistance changes that were converted into electrical signals. Simultaneously, the conductive f‐PANi network restored electrical conduction across fibrotic regions, enhancing calcium transient velocity and improving electrophysiological performance. This enabled continuous monitoring and therapeutic modulation of infarcted myocardium without external stimulation (Figure 9e).

Given the inherent dynamic nature of the biological tissue environment due to constant changes in temperature, pressure, strain, and pH, bioelectronic systems integrating two or more sensing modalities are vital for comprehensive monitoring and precise therapeutic intervention.^[^ ^157^ ^]^ In this regard, an eutectogel‐based multimodal sensor designed for ultrasensitive and simultaneous monitoring of pressure and temperature from nostril airflow was recently reported (Figure 9f).^[^ ^158^ ^]^ Eutectogels are soft, stable, and self‐supporting materials composed of deep eutectic solvents (DES) embedded within a 3D polymer network, combining low volatility, high ionic conductivity, and tunable hydrogen‐bond interactions, while their dynamic DES‐polymer interactions confer self‐healing capability, enhancing elasticity, durability, and enabling sustainable, long‐lasting bioelectronic devices.^[^ ^159^, ^160^ ^]^ In this work, the eutectogel was synthesized via physical crosslinking of poly(N‐Acryloyl 2‐Glycine), tannic acid, and hydroxyapatite in a deep eutectic solvent composed of choline chloride and ethylene glycol. This composition provided the material with low modulus, self‐healing, robust adhesion, and biocompatibility. Self‐healing was enabled by dynamic hydrogen and coordination bonding among poly(N‐Acryloyl 2‐Glycine), hydroxyapatite, and tannic acid (Figure 9g). The adhesive properties were attributed to a combination of multiple non‐covalent interactions, including hydrogen bonding, electrostatic interactions, van der Waals forces, and metal coordination (Figure 9h). Under applied pressure, variations in the distance between electrodes of the eutectogel sensor induced capacitance changes, whereas elevated temperatures enhanced ion dissociation and mobility, resulting in decreased resistance. Stimulus decoupling was achieved by leveraging the selective sensitivity of capacitance to pressure and resistance to temperature within defined operating ranges. In practical demonstrations, the sensor accurately captured pressure and temperature signals from nostril airflow, mouth breathing, chest and abdominal movements, as well as pulse rate, highlighting its efficacy for diagnosing obstructive sleep apnea syndrome (Figure 9i).

Despite the excellent adhesion, self‐healing, and sensing capabilities demonstrated by the aforementioned systems, they lack self‐degrading functionality. As a result, implantable devices would require secondary surgical removal at the end of their service life, and wearable or non‐implantable systems would contribute to e‐waste.^[^ ^161^, ^162^ ^]^ To address this drawback, a stretchable and biodegradable self‐healing conductive composite has been developed for application in advanced bioelectronic systems.^[^ ^33^ ^]^ This system integrated a self‐healing conductive composite (SH‐CC) with a self‐healing elastomeric substrate (SH‐PLCL), enabling robust adhesion, autonomous self‐healing, and full biodegradability (Figure 9j). The SH‐CC consisted of PEDOT:PSS doped with polyethylene glycol, glycerol, and ionic liquid to enhance stretchability, conductivity, and autonomous self‐healing. The SH‐PLCL was synthesized using PLCL‐diol, IPDI, and disulfide‐based chain extenders. This imparted autonomous self‐healing via disulfide metathesis and hydrogen bonding (Figure 9k), while also ensuring full biodegradability under physiological conditions. Robust interfacial adhesion was achieved due to the chemical interactions of interpenetrating polymer chains and the formation of hydrogen bonds between SH‐CC and SH‐PLCL (Figure 9l). Biodegradation occurred through hydrolytic cleavage of ester bonds in the PLCL backbone, resulting in dissolution in PBS, thereby eliminating the need for surgical removal in implantable applications (Figure 9m). To demonstrate practical application, a multifunctional self‐healing electronic web composed of SH‐CC and SH‐PLCL were integrated with electrodes and a strain gauge and implanted onto a rat urinary bladder for real‐time physiological monitoring and modulation (Figure 9n). It recorded EMG signals to detect bladder voiding events, delivered electrical stimulation to induce urination, and monitored bladder expansion via the capacitive strain gauge. This highlighted the system's capability for continuous physiological monitoring and stimulation of soft, deformable organs (Figure 9n). While integrating a single auto‐POF can provide specific advantages such as self‐healing, self‐sensing, or biodegradability, integrating multiple frameworks enables the realization of truly multifunctional, self‐sustaining bioelectronic systems.

## 7 Conclusions, Challenges, and Perspectives

### 7.1 Conclusions

Integrating auto‐POFs with plastic bioelectronics represents a transformative advancement in the design and development of tissue‐interface technologies. By incorporating intrinsic functionalities such as self‐protection, self‐healing, self‐adhesion, self‐degradation, and/or self‐sensing directly into the polymer matrix, enables self‐adaptive, stable, and long‐term continuous operation in dynamic biological environments.^[^ ^163^, ^164^ ^]^ Auto‐POFs‐integrated plastic bioelectronic systems stand at the forefront of intelligent device design, facilitating the performance of complex tasks autonomously without the need for external intervention. Applications span from implantable neural interfaces to wearable health‐monitoring devices. As discussed in this review, significant research progress has been made in developing plastic bioelectronic systems with diverse device architectures and operational mechanisms, capable of performing various autonomous functionalities that emulate biological processes. Notably, a critical realization emerging from recent research is that the synergistic integration of multiple autonomous frameworks within a single polymer system is essential for achieving truly multifunctional, self‐sustaining bioelectronic performance. For instance, coupling self‐healing with self‐degradation not only extends device lifespan but also ensures safe elimination once healing capability is diminished or functionality is no longer required. Similarly, integrating self‐adhesion and self‐sensing enables intimate tissue‐device interfaces for precise physiological signal acquisition and therapeutic interventions. Such multi‐framework architectures are essential for the advancement of next‐generation tissue‐interfaced bioelectronics, facilitating autonomous operation, real‐time physiological monitoring, and safe biointegration without external control or surgical removal. As a result, these systems are inherently suited to the dynamic, often unpredictable conditions encountered in biological environments, offering promising solutions to meet the challenges of both wearable and implantable applications.

### 7.2 Challenges

Despite these promising developments, several key challenges remain in the design and implementation of auto‐POFs‐integrated plastic bioelectronic devices and systems, necessitating continued research efforts. Here, we propose several key areas for future investigation.

#### 7.2.1 Multiple Adhesion‐Detachment Cycles

Reversible adhesion is essential for devices intended for repeated application. Auto‐POF systems must enable not only selective but also repeatable adhesion and detachment under physiological conditions, with consistent performance across tissue types. Fine‐tuning adhesion strength and minimizing post‐detachment residue formation, especially in dynamic or wet environments, remains a key challenge.

#### 7.2.2 Balancing Mechanical Robustness and Self‐Healing Efficiency

Self‐healing materials typically utilize dynamic covalent or non‐covalent bonds (e.g., hydrogen bonding, disulfide metathesis, and metal coordination) to enable reversible reconstruction of damaged networks. Owing to their relatively low binding energies, these dynamic bonds can act as energy dissipation sites under mechanical stress, thereby enhancing the material's toughness to some extent. Increasing their content to boost healing efficiency often compromises mechanical strength, elastic modulus, and fatigue resistance. For bioelectronic applications requiring long‐term mechanical stability, future auto‐POF systems must integrate highly robust mechanical frameworks while maintaining efficient self‐healing behavior.

#### 7.2.3 Improving Healing Kinetics and Response Time

Real‐time biosensing requires systems capable of rapidly recovering electrical and mechanical functionalities upon damage in complex, dynamic environments.^[^ ^165^ ^]^ However, many current self‐healing materials exhibit relatively slow recovery, particularly under physiological or extreme pH conditions. These limitations hinder the timely recovery of both mechanical integrity and electrical functionality, thereby compromising the performance of the material and the device. Accelerating the healing process and improving efficiency under physiological conditions remain critical for ensuring reliable long‐term operation.

#### 7.2.4 Programmable Degradation

For implantable devices, maintaining functionality for a defined period of time before degradation is vital. However, unregulated degradation often leads to premature loss of mechanical and electrical performance, compromising signal accuracy and reliability in clinical settings. Future research should focus on integrating programmable degradation mechanisms that preserve functionality during operation and ensure complete, safe elimination after service life. Addressing this challenge may involve integrating self‐reporting components that signal degradation status.

#### 7.2.5 Long‐Term Stability and Sensing Reliability

Auto‐POFs must maintain stable performance in dynamic biological environments involving repeated mechanical deformation, hydration changes, temperature fluctuations, and biochemical noise. Polymeric self‐healing materials generally exhibit faster recovery compared to inorganic counterparts; however, in scenarios such as real‐time biosignal monitoring or prolonged cyclic damage, even this recovery rate may be insufficient, and incomplete restoration over repeated healing cycles can accumulate, ultimately compromising signal fidelity and device reliability. In addition, conductive polymers such as PEDOT:PSS suffer from gradual conductivity loss under humid conditions, while hysteresis can induce signal distortion and delayed responses,^[^ ^166^ ^]^ thereby reducing diagnostic accuracy. Future research should therefore focus on designing polymer systems that not only ensure long‐term electrical and mechanical stability but also achieve accelerated healing responses tailored to the temporal demands of specific applications and reduced hysteresis. Such properties are essential for enabling high‐fidelity, long‐term biosignal monitoring in wearable and implantable devices.

#### 7.2.6 Minimizing Interference Among Integrated Functionalities

When multiple autonomous capabilities, such as self‐healing, self‐sensing, self‐adhesion, and self‐degradation, are integrated within a single system, functional interference may arise. To mitigate such trade‐offs, modular material architectures, selectively responsive elements, and multiscale structural design could support coordinated operation. In addition, advanced strategies, including spatiotemporal decoupling and dynamic feedback regulation, may further reduce crosstalk and ensure stable performance in complex biological environments. A close example implementation of spatiotemporal de‐synchronization to reduce crosstalk is a layered patch that i) localizes robust adhesion to the device perimeter; ii) fast disulfide exchange in the conductor to close microcracks in seconds; and iii) defers end‐of‐life via a slow‐hydrolyzing PLCL encapsulant. Spatial decoupling is reinforced by routing stimulation electrodes on a non‐adhesive island with serpentine interconnects, isolating them from the sensing core. Together, patterned layers (rim adhesion, islands) and staggered kinetics (seconds‐scale healing vs weeks‐scale degradation) minimize interference among adhesion, healing, sensing, and degradation.^[^ ^33^ ^]^

#### 7.2.7 Suitability for Clinical Medical Devices

One of the critical considerations for applying auto‐POFs in real medical devices is their performance under acute versus chronic conditions. In acute applications, such as intraoperative monitoring or temporary wound dressings, rapid self‐healing and strong yet reversible adhesion can provide distinct advantages. However, chronic applications require long‐term stability, resistance to fibrotic encapsulation,^[^ ^167^ ^]^ and minimal signal degradation. In particular, fibrotic activation and scar tissue formation in chronic implants can increase impedance or reduce the SNR.^[^ ^168^ ^]^ Therefore, to ensure reliable long‐term functionality, strategies such as incorporating anti‐fibrotic surface coatings,^[^ ^169^ ^]^ implementing adaptive calibration protocols,^[^ ^170^ ^]^ and designing hybrid frameworks^[^ ^171^ ^]^ are required.

#### 7.2.8 System Integration and Translational Barriers

Auto‐POFs also face challenges in system integration and clinical translation. Many materials are incompatible with conventional cleanroom processes involving acids, bases, or high temperatures, creating difficulties in large‐scale manufacturing and packaging. Furthermore, regulatory approval, good manufacturing practices (GMP)‐compliant production, and long‐term safety validation remain significant translational hurdles. Addressing these issues will require material innovations that not only enhance intrinsic performance but also meet process compatibility and regulatory requirements, thereby bridging the gap between laboratory advances and clinical application.

### 7.3 Perspectives: Opportunities and Outlook for Auto‐POFs

Beyond addressing existing challenges, several emerging opportunities are anticipated to accelerate the progress of auto‐POF research. One promising direction is the design of hybrid architectures that integrate auto‐POFs with inorganic nanomaterials such as 2D materials, ultrathin silicon, or metal oxides. These systems could improve electrical stability and enhance long‐term performance for implantable and wearable applications. Another important avenue is the use of artificial intelligence (AI)‐ and machine learning–assisted design, which can accelerate the discovery and optimization of polymers with tailored healing kinetics, conductivity retention, and degradation profiles. By leveraging machine learning–driven predictive models, high‐throughput screening, and data‐guided molecular simulations, it will become possible to rapidly identify novel polymer chemistries and optimize multi‐framework integration with unprecedented efficiency. Such AI‐guided strategies could, for instance, uncover optimal trade‐offs between mechanical robustness, healing kinetics, and sensing stability, which are otherwise difficult to balance experimentally. Moreover, coupling AI‐driven design with automated polymer synthesis and device fabrication platforms will establish closed‐loop pipelines capable of developing self‐regulating bioelectronic systems in an accelerated and adaptive manner. This convergence of intelligent computation with autonomous material design opens a pathway toward next‐generation plastic bioelectronics that are not only self‐sustaining but also self‐evolving, enabling personalized, adaptive, and resilient interfaces with dynamic tissues. In addition to these directions, related fields such as self‐powered materials^[^ ^172^, ^173^ ^]^ and mechanically adaptive polymers^[^ ^174^ ^]^ (e.g., shape‐memory polymers that soften near 37 °C) are acknowledged as highly relevant but lie outside this scope. Self‐powered systems address energy autonomy, not interface autonomy; their mechanisms and metrics (power density, rectification) are orthogonal to the interface‐centric failure modes that auto‐POFs resolve. Likewise, shape‐memory/phase‐transition systems primarily deliver programmed macroscopic mechanics (deployment, softening) typically triggered thermally or by external scheduling. These fields hold promises for synergistic integration with auto‐POFs, and Comprehensive coverage of these domains requires a dedicated separate review.

In summary, advancing auto‐POFs for tissue‐interfaced bioelectronics will require addressing persistent challenges while also embracing emerging opportunities. Moreover, to overcome limitations in long‐term stability, multimodal integration, and programmable degradation will demand interdisciplinary approaches that combine polymer chemistry, materials science, biomedical engineering, and device physics. At the same time, new directions such as hybrid polymer‐inorganic architectures, and AI‐assisted material design highlight the potential for transformative innovations. To support future research directions, we additionally provide two summary tables that offer representative benchmarks and performance metrics. **Table** **1** outlines the mechanical properties of native tissues and practical targets for interfacial adhesion and toughness, while **Table** **2** summarizes key characteristics of auto‐POF‐based devices across different tissue‐interfaced applications. These references serve to guide rational material and device design. Together, these strategies position auto‐POFs as foundational components in the development of future wearable and implantable systems, ultimately enabling autonomous, tissue‐integrated electronic platforms that operate seamlessly in harmony with the human body.

**Table 1:** Young's modulus of common biological tissues and practical target ranges for interfacial adhesion and toughness.

Target tissue	Tissue modulus	Guideline for auto‐POFs	Refs.
Skin (Dermis)	8–74 kPa	Adhesion strength: 0.6 MPa (thickness: 7 µm) Interfacial toughness: 10–20 J m^−2^ Interfacial toughness: 670 J m^−2^ (hemostatic patch)	[85, 175, 176, 177, 178]
Skin (Hypodermis)	2–8 kPa		
Heart	8–15 kPa	Adhesion strength: 4.84–13.65 kPa Interfacial toughness: 166.2–443.4 J m^−2^	[156, 179]
Vessel (vascular)	0.2–0.6 MPa	Interfacial toughness: 1300–1400 J m^−2^	[175]
Liver	4.0–6.5 kPa	Interfacial toughness: 1116 J m^−2^	[180, 181, 182, 183]
Intestine	20–40 kPa	Adhesion strength: 38.7–24.1 kPa (underwater for 0.5–24 h)	[184]

**Table 2:** Summary of representative target performance metrics of tissue‐interface devices.

Target application	Guideline for auto‐POFs		Refs.
	Mechanical property	Performance	
Skin‐ e‐skins, wearable patches, ECG, EMG	Thin or soft polymer (modulus: 10–100 kPa)^[^ ^185^, ^186^, ^187^ ^]^	S: 48.1–5.77 kPa^−1^ (0–135 kPa)	[189]
		S: 18.1–1.0 kPa^−1^ (0–95 kPa)	[60]
		GF: ≥ 2.19, SNR: 8.91–40 (ECG) and 5.63–36 (EMG)	[190, 191]
Neural interfaces, soft electrodes	Modulus: < 10 kPa^[^ ^188^ ^]^	Power density: 0.85µW cm^−3^	[137]
		SNR: 60–100 (ECG)	[193]
		Activation of rat limb muscle at 2.5 V with 1.38 N	[4]
Hydrogel for implants, hemostatic patches	Shear strengths: 50–100 kPa^[^ ^192^ ^]^		
Soft, tissue‐interfaced pH sensing	Strain: ≥ 100%^[^ ^194^ ^]^	S: ≥ 40–60 mv pH^−1^	[194, 195]
Soft, tissue‐interfaced temperature sensing	Strain: ≥ 30%^[^ ^141^ ^]^	S: ≥ 0.95%·^°^C^−1^ (20– 40 °C)	[145]

S: sensitivity; SNR: signal‐to‐noise ratio; GF: gauge factor

## Conflict of Interest

The authors declare no conflict of interest.
